# The Effects of Traditional Chinese Exercise in Patients with Chronic Obstructive Pulmonary Disease: A Meta-Analysis

**DOI:** 10.1371/journal.pone.0161564

**Published:** 2016-09-02

**Authors:** Xiaotian Luo, Jifeng Zhang, Rachel Castelberg, Tao Wu, Pengming Yu, Chengqi He, Pu Wang

**Affiliations:** 1 Department of Rehabilitation Medicine Center, Sichuan University, West China Hospital, Chengdu, Sichuan, PR China; 2 Department of Key Laboratory of Rehabilitation Medicine in Sichuan, Chengdu, Sichuan, PR China; 3 Department of General surgery Medicine, Datong Second People’s Hospital, Datong, Shanxi, PR China; 4 Department of Physical Therapy, University of North Texas Health Science Center, 3500 Camp Bowie Blvd, Fort Worth, United States of America; Universite de Bretagne Occidentale, FRANCE

## Abstract

**Background:**

Chronic obstructive pulmonary disease (COPD) is a major public health problem worldwide. However, several studies that have assessed the role of traditional Chinese exercise in the management of this disease include broad variations in sample sizes and results. Therefore, this meta-analysis was conducted to assess the effects of traditional Chinese exercise on patients with COPD.

**Methods:**

Two investigators independently identified and extracted data from selected articles. A computerized search of electronic databases through August 2015 was conducted. Mean differences (MDs) and 95% confidence intervals (CIs) were calculated to analyze the combined data. The methodological quality was evaluated using the Cochrane risk-of-bias tool. Heterogeneity was assessed with the I^2^ test.

**Results:**

Ten randomized, controlled trials (RCTs) involving 622 patients met the inclusion criteria. There were significant improvements in the 6-minute walking distance test (6 MWD;MWD = 12.10 m; 95% CI, 7.56–16.65 m; p<0.001); forced expiratory volume in one second (FEV1% predicted; WMD = 9.02; 95% CI, 6.80–11.23; p<0.00001); forced expiratory volume in 1 second/forced vital capacity (FEV(1)/FVC) ratio (Tiffenau Index; WMD = 6.67; 95% CI, 5.09–8.24; p<0.00001); and quality of life, as evaluated by the Chronic Respiratory Disease Questionnaire (CRDQ; WMD = 0.85 score; 95% CI, 0.52–1.18; p<0.00001).

**Conclusions:**

Traditional Chinese exercise could provide an effective alternative method for managing COPD. Larger and higher-quality trials are required.

## Introduction

Chronic obstructive pulmonary disease (COPD) involves airflow limitations that are not fully reversible. It develops progressively and causes serious harm to human health. COPD is currently ranked 12th in global disease incidence rate and 4th in worldwide causes of death [1,2]. In China, COPD, which is one of the most common chronic respiratory diseases, is also a serious threat to people's health. A cross-sectional survey in China showed a COPD prevalence of 8.2% in people >40 years of age [3]. The effects of COPD on physical and emotional health may lead to disability and impaired mood, which in turn influences patients’ quality of life. In addition, these physical and psychosocial constraints can increase the need for family and social support.

Traditional Chinese exercise (TCE) is an ancient Chinese system of gentle, self-healing exercise designed to train the functional integrity of and enhance the vitality of the energy called Qi. This vital energy flows through all of the organ systems and tissues of the body via channels called meridians and collaterals. When Qi is rich, free flowing, and in balance, a person usually has good health and longevity. Therefore, traditional Chinese exercise may be a suitable exercise style for patients with COPD, as it provides mild to moderate aerobic activity and strength training of the lower extremities, unsupported upper limbs, and core. It also addresses breathing, respiratory muscle training and stress control, which are important aspects of COPD management. In recent years, the effectiveness of traditional Chinese exercises for rehabilitating stable COPD patients has received increasing recognition and attention. Currently, traditional exercises used by stable COPD patients include Tai Chi, Liu Zijue, Wu Qinxi and Ba Duanjin. Research has shown that these traditional exercises are not only simple but have a positive impact on rehabilitation by improving the stability of lung function, movement endurance and quality of life in patients with COPD [4–6].

However, the sample sizes of individual trials have been small, and the results have been mixed and inconclusive. To date, no systematic reviews with meta-analyses have been performed to specifically examine the effects of traditional Chinese exercises for people with COPD. Using the meta-analysis method, this study undertook a comprehensive quantitative analysis of traditional exercises for patients with stable COPD to provide scientific guidance regarding this treatment method.

## Methods

### Data Sources and Searches

Databases were searched from their inception to August 2015. The search included MEDLINE, EMBASE, CENTRAL, the Cochrane Library, the Chinese BioMedical Literature Database, the China National Knowledge Infrastructure (CNKI), the Chinese Medical Database (CMD), Taiwanese academic online databases, Google Scholar and ClinicalTrials.gov. The following search terms were used: traditional exercise or traditional fitness exercise or traditional exercise therapy or mind-body exercise or Qigong or health Qigong or Tai Chi or Tai Ji or Tai Chi exercise or Taichi Qigong or Liu Zijue or Ba Duanjin or Wu Qinxi or Yi Jinjing and chronic obstructive pulmonary disease or COPD. Two raters performed the data searches (L.X. and Z.J.). Human subjects and randomized controlled trials (RCTs) were required for inclusion. There was no language restriction. The literature retrieval and the identification of all potentially related articles (including unpublished articles, meta-analyses, and relevant articles from personal contacts who are experts in this field) and international guidelines were performed manually.

### Inclusion Criteria

The inclusion criteria conformed to the PICOS approach, as described below.

#### Patients

According to the Global Initiative for Chronic Obstructive Lung Disease (GOLD) [1] criteria for diagnosing COPD, patients should be in a stable phase of the disease with no acute exacerbations for six months prior to the test.

#### Intervention

The intervention time was longer than 6 weeks. The experimental group only participated in traditional exercises, such as mind-body exercises, Qigong, Tai Chi, Tai Ji, Taichi Qigong, Liu Zijue, Ba Duanjin or Wu Qinxi and Yi Jinjing. The control group received conventional therapy, including drug treatments and routine health guidance.

#### Outcome measurements

From the perspective of rehabilitation, effective interventions should not only reduce impairments, but also improve activity and participation. We focused on used commonly outcome measures reflecting function and activity in people with COPD.

The primary outcomes was to investigate the effect of functional performance:

1. Mobility (e.g. Six-minute walk test,)
2. Lung Function (eg. forced expiratory volume in one second [FEV1% predicted] and forced vital capacity rate in one second [Tiffenau Index])

The secondary outcome was to evaluate the quality of life:

1. Quality of life (eg. dyspnea, emotion, fatigue, and mastery).
2. Adverse events.

#### Study design

RCTs examining rehabilitation treatments that included traditional exercises in stable COPD patients.

### Exclusion criteria

Trial were excluded if they (1) used Qigong and Ba Duanjin involving breathing techniques and meditation but no physical activity; (2) were observational studies, case series, or case reports; or (3) had a score ≤2 for methodological quality, which we evaluated using the Jadad scale.

### Data Extraction and Analysis

All studies were reviewed, and the data were extracted independently by two raters (X.T.L. and J.F.Z.). Disagreements were resolved by seeking the opinion of a third rater (X.P.L.). For each eligible study, the following information was extracted and recorded: (1) first author’s name, (2) publication year, (3) intervention and control group information, (4) intervention duration, (5) sample size, (6) participants’ demographic characteristics, and (7) primary and other outcome measurements. The mean changes in outcome measurements compared with baseline were used to assess the differences between the intervention and control groups. Pooled effect sizes and 95% confidence intervals (CIs) were calculated using Cochrane Collaboration software (Review Manager [RevMan], version 5.3 for Windows, downloaded from http://ims.cochrane.org/revman/download). Mean differences (MDs) were used for continuous data. The x^2^ test and the Higgins I^2^ test were used to assess heterogeneity.

### Quality and Risk of Bias Assessments

The Jadad scale was used to assess the methodological quality of each study [7]. A score ≤2 indicates low quality, and a score ≥3 indicates high quality [8]. The Cochrane Handbook for Systematic Reviews of Interventions (RevMan version 5.3, the Cochrane Collaboration, 2011) was used to evaluate the risk of bias. Two authors (L.X. and Z.J.) subjectively reviewed all articles and assigned a value of ‘high’, ‘low’, or ‘unclear’ based on the following: (a) selection bias (whether there was adequate generation of the randomization sequence and whether the allocation concealment was satisfactory); (b) blinding (i.e., performance bias and detection bias, including whether there was blinding of the participants, personnel, and outcome assessments); (c) attrition bias (whether incomplete outcome data were sufficiently assessed and addressed); (d) reporting bias (whether there was evidence of selective outcome reporting); and (e) other biases (whether the study was free of other problems that could increase the risk of bias). Any disagreements were resolved by discussion and consensus. To improve accuracy, a third investigator (X.P.L.) was consulted when any disagreement emerged. Any analytical data missing from the primary reports were requested from the authors. When the same study was reported in multiple publications, we used the most recent study to avoid duplicating information.

### Statistical Analysis

RevMan software, version 5.3, was used to analyze the data from the included studies. For continuous outcomes, MDs were used to calculate the difference between means; 95% confidence intervals (CIs) were treated as an effective indicator when all measurements were estimated from each study; and fixed effects and random effects models were used to pool data across studies [9]. As a quantitative measurement of inconsistency across studies, heterogeneity was tested using the I^2^ statistic. Studies with an I^2^ statistic of 25% to 50% were considered to have low heterogeneity, those with an I^2^ statistic of 50% to 75% were considered to have moderate heterogeneity, and those with an I^2 ^statistic >75% were considered to have high heterogeneity [10]. When the I^2^ statistic was >50%, sensitivity and subgroup analyses were performed to identify the potential sources of heterogeneity. Pre-specified sensitivity analyses were performed by removing one study at a time and determining the influence of a single article on the overall pooled estimate. The potential publication bias for each analysis was assessed using a funnel plot. When the number of articles included in one analysis was limited (less than 10), publication bias was not assessed. Statistical significance was indicated by a P value <0.05. This study applied an intention-to-treat (ITT) analysis and followed the Preferred Reporting Items for Systematic Reviews and Meta-Analyses (PRISMA) statement [11].

## Results

### Literature Search Results

**Fig 1:**
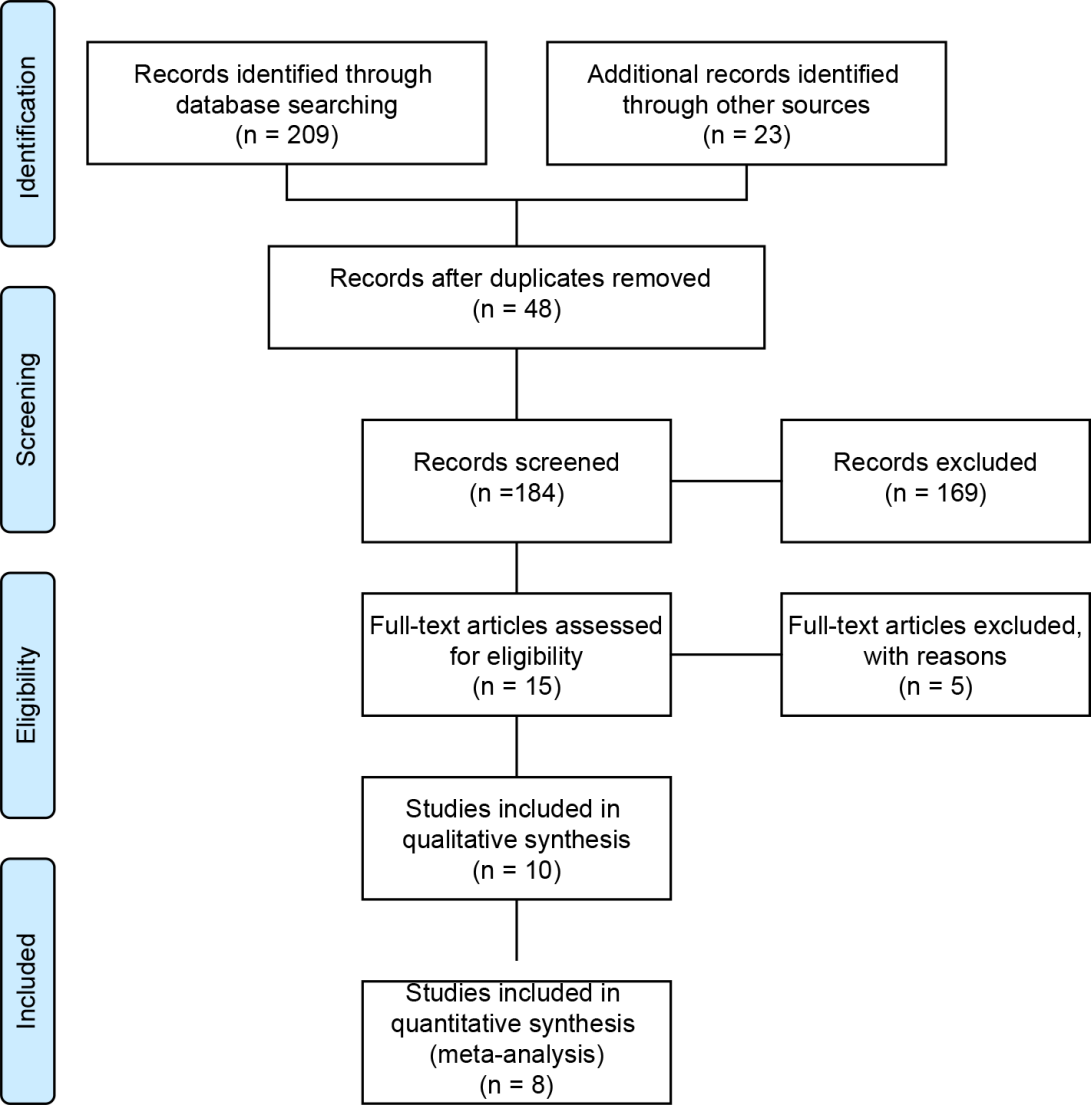
Search strategy and flow chart of the screened, excluded and analyzed articles.

Through database searches, 232 potentially relevant articles were identified. Forty-eight duplicates were removed, and 169 reviews and unrelated articles were excluded based on the titles and summaries. Finally, based on the full texts of the remaining 15 articles, 10 (nine published in English and one published in Chinese) met the inclusion criteria, and 8 of those were included in the meta-analysis (Fig 1).

### Trial Characteristics

Ten RCTs [12–20] were selected for this meta-analysis. Nine [4,13–19] were published in English, and one was published in Chinese [12]. Three RCTs that enrolled the same population or involved the same trials were pooled in our meta-analysis and data from these three articles were combined because some important outcomes were included separately in the three RCTs [18–20]. In addition, two ongoing RCTs included protocol information for the same population [4,14] and were combined. The studies were published between 2010 and 2014. The sample size ranged from 5 to 206 (total, 622). Five RCTs reported results from the 6 MWD [12,13,15,16,18]. Quality of life was evaluated with the CRDQ in three RCTs [13–15]. Three RCTs [12,16,17] reported FEV1, and three [12,15,17] reported FVC. Follow-up periods ranged from 6 to 48 weeks, and the exercise time lasted from 30 to 60 min. The type of intervention was Tai Chi or Qigong in 5 RCTs[12,14,15,16,17], short-form sun style in 1 RCT [4], Ba Duanjin in 1 RCT [13], and13 movements of Breathing Regulating Tai Chi Qigong (13BRTCQ) in 3 RCTs [18,19,20]. The interventions provided for the control or comparison groups in all included trials were education, breathing techniques, and walking with or without breathing techniques. The patients in all trials received the standard pharmacological treatment in addition to the experimental intervention. The characteristics of the included trials are listed in Table 1.

**Table 1:** Characteristics of the randomized controlled trials included in the meta-analysis.

Study [ref]	Study design	Jadad score	Patient no. (M/F)	Age, mean (I/C)	FEV1 (% of predicted)	Study group (n)	TC form or style	Protocol	Adherence/adverse effects	Outcomes
Du 2013 [12]	RCT	3	112 (70/42)	65.24/64.48	TC (73.36±6.33), exercise (74.28±7.39), control (72.97±6.46)	TC (36), exercise (38), control (38)	24-short form TC	12 weeks * 2 times/day, 30 min/per time	100%/none	FEV1, FEV1/FVC (%), MVV (L/min), SaO_2_(%), 6 MWD (m), CAT
Lorna 2014 [4]	RCT	4	42 (22/20)	63.1/62.0	TC (102±23), control (102±22)	TC (21), control (21)	Short-form sun style	12 weeks * 1 time/day, 50 min/per time	100%/none	ISWT, ESWT, MPPB, HRQoL, HADS, FPI
Bobby 2011 [13]	RCT	4	80 (71/9)	71.7/73.1	TC (37.13±2.22), control (36.75±2.11)	TC (40), control (40)	Ba Duanjin	24 weeks * 4 times/week, 45 min/per time	67%/none	6 MWD, HRQoL
Regina 2013 [14]	RCT	4	42 (23/19)	73/75	TC (59±16), control (63±14)	TC (22), control (20)	TC	12 weeks * 2 times/day, 60 min/per time	86%/none	ISWT, ESWT, MPPB, HRQoL, VO_2_, VCo_2_
Gloria 2010 [15]	RCT	3	10 (6/4)	65/66	TC (53±7), control (47±7)	TC (5), control (5)	TC	12 weeks * 2 times/day, 60 min/per time	100%/none	6 MWT, UGT, HRQoL
Niu 2014 [16]	RCT	5	40 (37/3)	61.3/59.7	TC (41.9±5.50), control (43.7±5.16)	TC (20), control (20)	TC	24 weeks * 7 times/week, 50 min/per time	95%/none	6 MWD, FEV1, FEV1% pre, TwPes
Liu 2012 [17]	RCT	3	132 (91/41)	61.82/62.2	HQG (74.43±12.93), PR (75.31±12.84), control (75.31±13.79)	HQG (51), PR (32), control (35)	Qigong	24 weeks * 3 times/week, 60 min/per time	89%/none	6 MWD, HRQoL
Chan 2010 [19], 2013 [18,20]	RCT	5	206 (188/18)	71.7/73.6	TC (50.1±21.8), exercise (56.4±25.6), control (55.1±23.3)	TC (70), exercise (69), control (67)	13BRTCQ	12 weeks * 2 times/week, 60 min/per time	86%/none	6 MWD, dyspnea, fatigue, HRQoL, MSPSS-c, FVC, FEV1, BORG, SCALE, SaO_2_

Legend: RCT, randomized controlled trial; M/F, male/female; TC, Tai Chi; I/C, intervention/control; FEV1, forced expiratory volume in one second; FVC, forced vital capacity; TCQ, Tai Chi Qigong; Ba Duanjin, eight-length brocade exercise; 6 MWD, 6-minute walking distance; HRQoL, health-related quality of life; CAT, COPD assessment test; ESWT, endurance shuttle walk test; FPI, functional performance inventory; HADS, Hospital Anxiety and Depression Scale; ISWT, incremental shuttle walk test; MPPB, modified physical performance battery test; VCo_2_, carbon dioxide production; VO_2_, oxygen consumption; TwPes: twitch esophageal pressure. There were no significant differences in the FEV1 (% of predicted) among groups at baseline according to the data reported in each article; 13BRTCQ, 13 movements of Breathing Regulating TCQ.

### Methodological Quality

**Fig 2:**
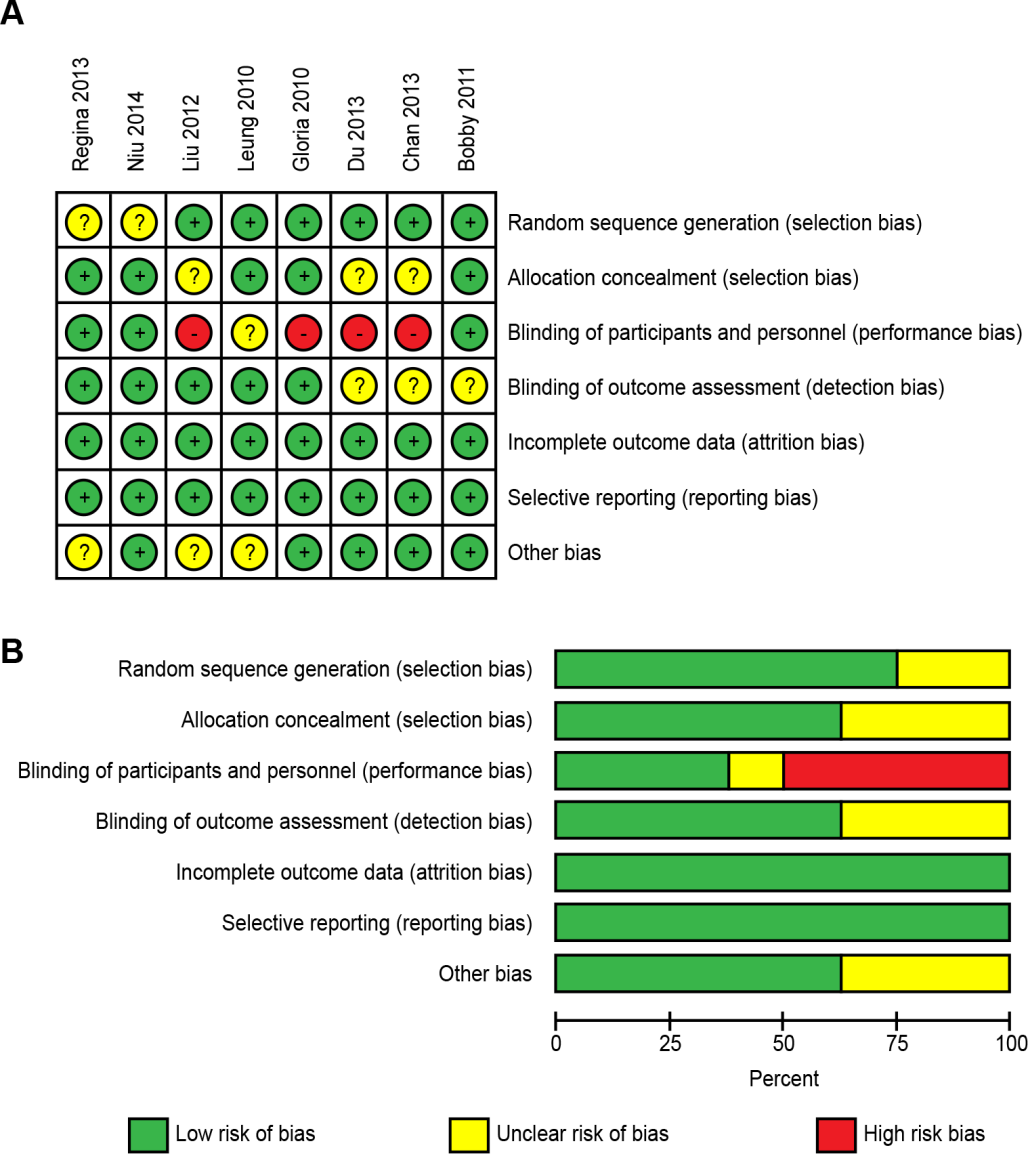
Risk-of-bias analysis. (A) Risk-of-bias summary: The authors’ judgments regarding each risk-of-bias item for each included study. (B) Risk-of-bias graph: The authors’ judgments regarding each risk-of-bias item presented as percentages across all included studies.

Two investigators (L.X.T. and Z.J.F.) agreed on the Jadad score for each study. The mean Jadad score of the included studies was greater than 3. All but two of the RCTs [14,16] described the methods of randomization, but only five [4,13–15] reported allocation concealment details. Five trials [4,14–17] mentioned the blinding of assessors for data collection. All of the articles reported complete outcome data. The risk-of-bias analysis is shown in Fig 2.

### Outcomes

#### 6 MWD

**Fig 3:**
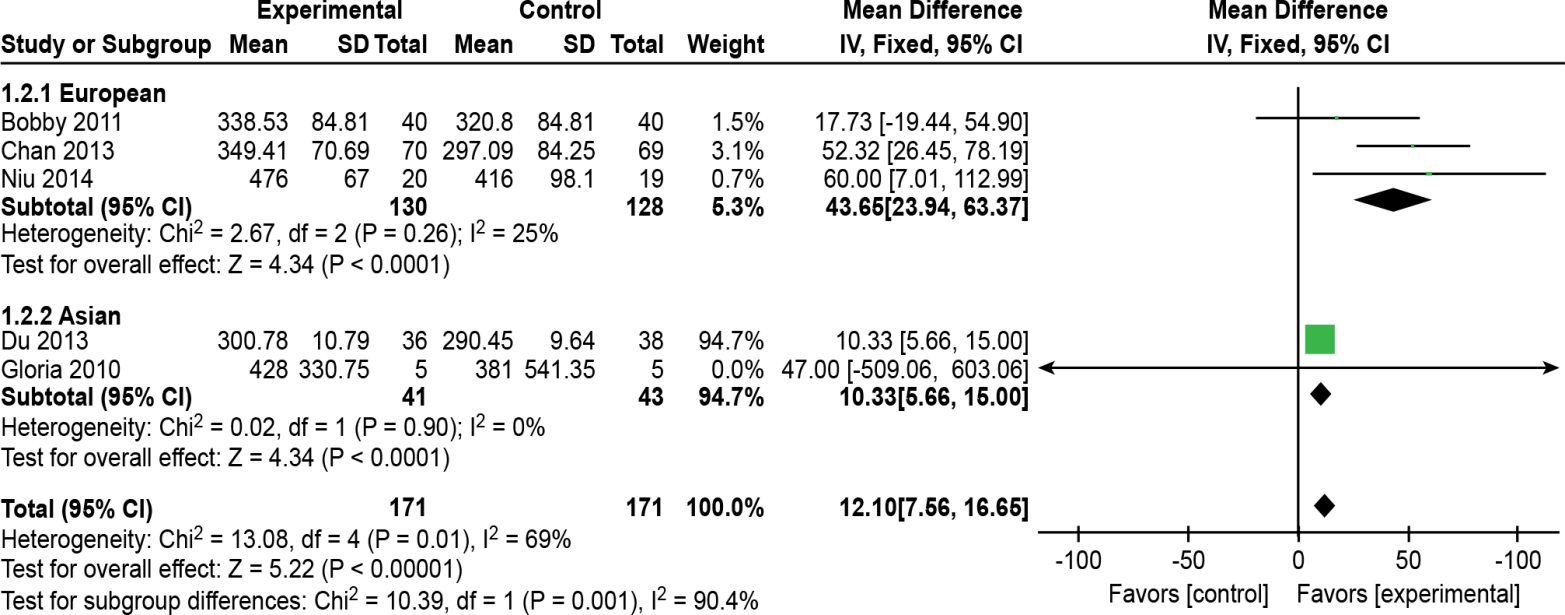
Effects of traditional Chinese exercise on 6-minute walking distance. The subgroup meta-analysis of RCTs evaluating the effects of traditional Chinese exercise on the 6 MWD using a fixed effects model.

Five of the 10 trials [12,13,15,16,18] (n = 314 patients) compared the distance walked during the 6 MWD for the Tai Chi or Qigong, Ba Duanjin and control groups. An unsuccessful attempt was made to contact the authors via e-mail to obtain the applicable data. Two trials [12,15] reported an improvement in the 6 MWD after 3 months of Tai Chi, Qigong, or Ba Duanjin compared with the control group. The other three trials [13,16,18] reported outcomes after 6 months of Tai Chi or Qigong. The five trials showed heterogeneity when they were pooled in a meta-analysis (P = 0.01, I^2^ = 69%). The five articles were read again in full, and their data collection processes were checked to confirm that there were no errors. The subgroup and sensitivity analyses of the data from the five trials showed that there was a difference in race. The Asian and European subgroups showed homogeneity, and the results are shown in Table 2. A random effects meta-analysis was performed to incorporate the heterogeneity among these five studies. A pooled effect size of five trials [12,13,15,16,18] showed that the TCE intervention was associated with a statistical improvement on the 6 MWD and could increase the length of the 6 MWD compared with the control intervention (MD, 12.10 m; 95% CI, 7.56–16.65 m; P<0.001; Fig 3).

**Table 2:** Sensitivity analyses of the 6 MWD and Tiffenau index, excluding low-quality trials.

Outcome	n (N)	MD (95% CI)	P value	I^2^ (%)	P_heterogeneity_
6 MWD					
All included trials [12,13,15,16]	342 (4)	12.10 [7.56, 16.65]	P<0.001	69%	P = 0.01
High-quality trials [13,16,18]	258 (3)	43.65 [23.94,63.37]	P<0.0001	25%	P = 0.26
Low-quality trials [12,15]	84 (2)	10.33 [5.66, 15.00]	P<0.0001	0	P = 0.9
FEV1/FVC					
All included trials [12,15,17]	170 (3)	4.85 [3.51,6.18]	P<0.00001	89%	P<0.00001
High-quality trials [12,15]	84 (2)	6.67 [5.09,8.24]	P<0.00001	0	P = 0.75
Low-quality trials [17]	86 (1)	0.08 [-2.47,2.63]	P = 0.95	--	--

Legend: 6 MWD, 6-minute walking distance; Tiffenau Index, forced expiratory volume in 1 second/forced vital capacity (FEV(1)/FVC) ratio; n, number of patients, N, number of trials.

#### FEV1 percentages predicted in Lung Function

**Fig 4:**
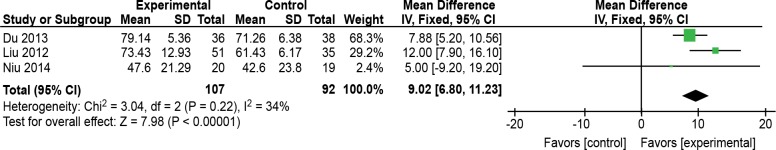
Effects of traditional Chinese exercise on forced expiratory volume in one second. Meta-analysis of RCTs evaluating the effects of traditional Chinese exercise on FEV1 using a fixed effects model.

Three of the 10 trials [12,16,17] (n = 199 patients) compared the FEV1 percentages predicted in the TCE intervention and control groups. One [16] of those articles could not be pooled directly in the meta-analysis because it did not provide data in the correct format. One trial [12] reported an improvement in the FEV1 percentage predicted after 3 months of Tai Chi or Qigong compared with the control intervention, and two other trials [16,17] reported the outcome after 6 months of Tai Chi or Qigong. These three trials showed homogeneity when they were pooled in a meta-analysis (P = 0.22, I^2^ = 34%). Therefore, the fixed effects meta-analysis was used to analyze the three studies. The pooled effect size of these three trials [12,16,17] showed that the TCE intervention was associated with a statistically significant improvement in the FEV1 percentage predicted and could increase COPD patients' lung function, as measured by the FEV1 percentage predicted, compared with the control group (MD, 9.02; 95% CI, 6.80–11.23; P<0.00001; Fig 4).

#### Tiffenau Index of Lung Function

**Fig 5:**
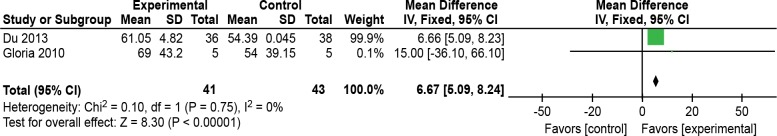
Effects of traditional Chinese exercise on the forced vital capacity rate in one second. A meta-analysis of RCTs evaluating the effects of traditional Chinese exercise on the Tiffenau Index using the fixed effects model.

Three of the 10 trials [12,15,17] (n = 169 patients) compared the FEV1 percentage predicted between the TCE intervention and control groups. One [15] of those articles could not be pooled directly in the meta-analysis because it did not provide the data in the correct format. One trial [12] reported that there was an improvement in the Tiffenau Index after 3 months of Tai Chi or Qigong compared with the control intervention, and the other two trials [15,17] reported the outcomes after 6 months of Tai Chi or Qigong. The three trials showed heterogeneity when they were pooled in a meta-analysis (P<0.0001, I^2^ = 89%). A sensitivity analysis was performed to explain the heterogeneity (Table 2). The Tiffenau Index effect size of one trial [15] was obviously greater than that of the other two trials, and those two trials showed homogeneity (P = 0.75, I^2^ = 0%) when the trial with the larger effect size was removed. Therefore, the fixed effects meta-analysis was used to analyze the two studies. The pooled effect size of the two trials [12,15] showed that the TCE intervention was associated with a statistically significant improvement in the Tiffenau Index and could increase COPD patients' lung function compared with the control intervention (MD, 6.67; 95% CI, 5.09–8.24; P<0.00001; Fig 5).

#### Quality of life

**Fig 6:**
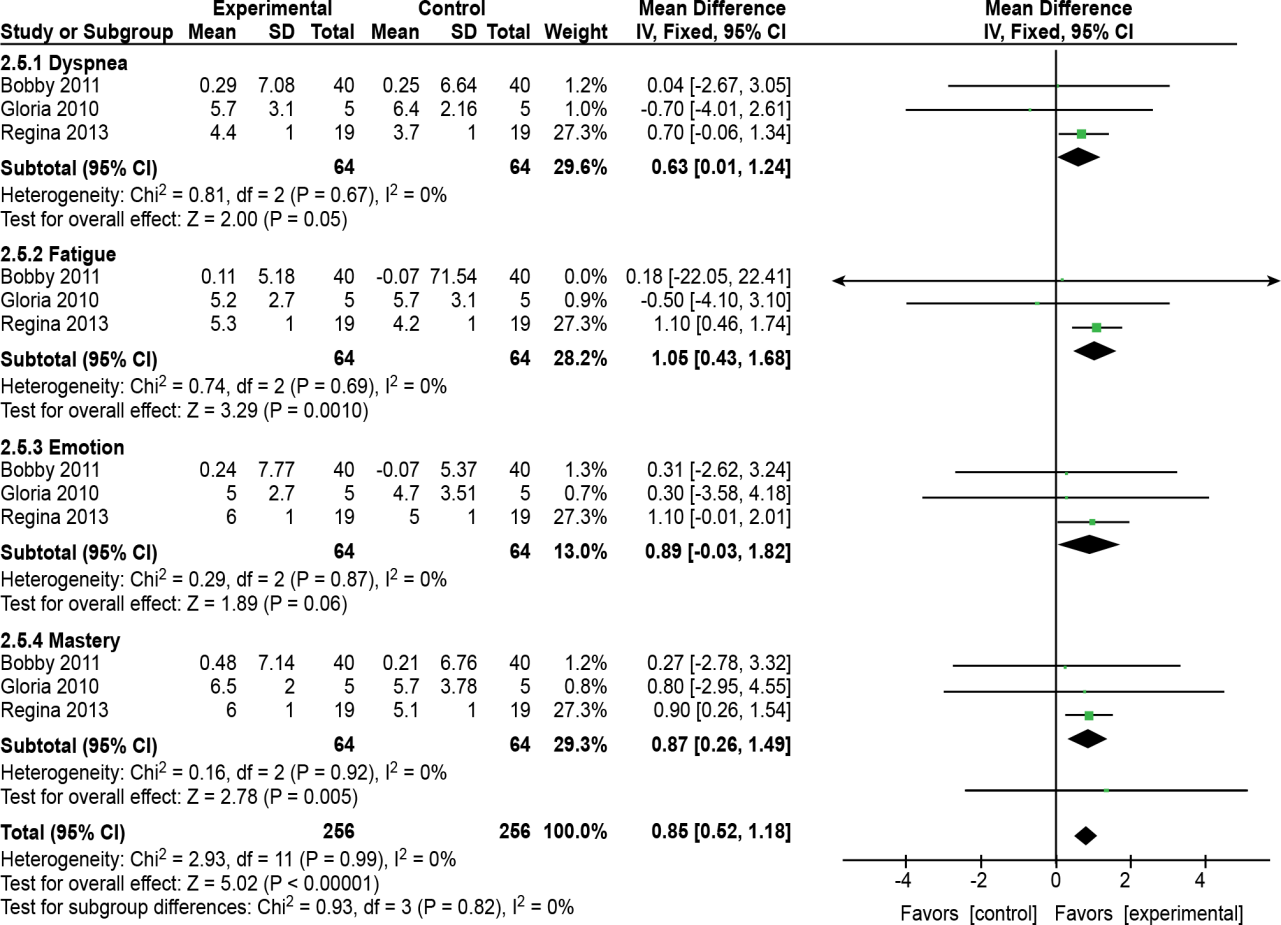
Effects of traditional Chinese exercise on health-related quality of life. **A meta-analysis of RCTs evaluating the effects of traditional Chinese exercise on health-related quality of life using a fixed effects model.**

Three of the 10 trials [4,13,14] (n = 128 patients) compared quality of life between the TCE intervention and control groups. One [4,13,14] of those articles could not be pooled directly in the meta-analysis because it did not provide data in the correct format. An unsuccessful attempt was made to contact the authors via e-mail to obtain the applicable data. Two trials [13,14] reported an improvement in quality of life after 3 months of Tai Chi or Qigong compared with the control intervention, and the other trial [4] reported the outcome after 6 months of Tai Chi or Qigong. The three trials showed homogeneity when they were pooled in a meta-analysis (P = 0.99, I^2^ = 0%). Subgroup analyses of the three trials' data were performed using a fixed effects meta-analysis. The total pooled effect size of the three trials [4,13,14] showed that the TCE intervention was associated with a statistically significant improvement in quality of life compared with the control intervention (MD, 0.85 score; 95% CI, 0.52–1.18; p<0.00001; Fig 6).

#### Dyspnea

The three trials showed homogeneity when they were pooled in a meta-analysis (P = 0.67, I^2^ = 0%). A fixed effects meta-analysis was performed. The pooled effect size of the three trials [4,13,14] showed that the TCE intervention was associated with a statistically significant improvement in dyspnea in terms of quality of life and could decrease dyspnea in COPD patients compared with the control intervention (MD, 0.63 score; 95% CI, 0.01–1.24; P = 0.05; Fig 6).

#### Fatigue

The three trials showed homogeneity when they were pooled in a fixed-effects meta-analysis (P = 0.69, I^2^ = 0%). The pooled effect size of the three trials [4,13,14] showed that the TCE intervention was associated with a statistically significant improvement in fatigue in terms of quality of life and could decrease fatigue in COPD patients compared with the control intervention (MD, 1.05 score; 95% CI, 0.43–1.68; P = 0.0001; Fig 6).

#### Emotion

The three trials showed homogeneity when they were pooled in a fixed-effects meta-analysis (P = 0.87, I^2^ = 0%). The pooled effect size of the three trials [4,13,14] showed that the TCE intervention was not associated with a statistically significant improvement in emotion in terms of quality of life and did not improve the emotion of COPD patients compared with the control intervention (MD, 0.89; 95% CI, -0.03–1.82; P = 0.06; Fig 6).

#### Mastery

The three trials showed homogeneity when they were pooled in a fixed-effects meta-analysis (P = 0.92, I^2^ = 0%). The pooled effect size of the three trials [4,13,14] showed that the TCE intervention was associated with a statistically significant improvement in mastery in terms of quality of life and could increase the mastery of COPD patients compared with the control group (MD, 0.87 score; 95% CI, 0.26–1.49; P = 0.005; Fig 6).

### Discussion

We performed a meta-analysis to assess the effects of traditional Chinese exercise included Taichi, Qigong, Liu Zijue, Ba Duanjin, Wu Qinxi, Yi Jinjing on patients with COPD. This is different from those articles [21–27] that searched the effectiveness of T’ai Chi and/or Qigong. We found that traditional exercises had rehabilitative effects on the 6 MWD, FEV1, Tiffenau Index, and CRDQ score compared with conventional treatment in stable COPD patients.

Decreased exercise tolerance is the one of main characteristics of COPD patients for several reasons. In COPD patients, an exercise endurance evaluation can increase understanding of the patient’s functional status, quality of life, and prognosis. Common clinical exercise endurance tests include the 6 MWD and incremental shuttle walk test (ISWT). In our study, we found that traditional Chinese exercise could increase the length of the 6 MWD compared with control conditions, but mean changes of 6 MWD were lower than the its minimum clinically important difference(MCID) (≥26 m) [28]. This result is consistent with some published studies. In healthy people, traditional Chinese exercise movements increase lower limb muscle strength [29–32], enhance knee and ankle proprioception [33], reduce station of body sway [34] and improve the speed of response to postural disturbances [35], which may reduce the risk of falls. A possible explanation for this might be that these styles of exercise training may increase the balance and coordination of COPD patients and reduce the patient's concerns about falling. This may improve the patient’s ability to move despite airflow obstruction, pulmonary over-inflation, and gas exchange impairment in the process of respiratory movement, which otherwise limit patients’ exercise capacity so severely that they are unable to perform rehabilitative exercises, which leads to an overall decrease in exercise ability [36]. These five trials showed heterogeneity when they were pooled in a meta-analysis, however, the subgroup and sensitivity analyses showed homogeneity between the Asian and European subgroups that may be related to differences in race. In these papers [21.22.24.25], there was no subgroup analysis of 6MWD about different ethnic groups.

Patients with COPD experience a decrease in exercise capacity and increased dyspnea, which is directly related to the damage to lung function. The most commonly used clinical indices of lung function are VC, FEV1, FEV1% predicted, the Tiffenau Index, MVV, FEV1% and the Tiffenau Index predicted value (%). These indices are important indicators of the degree of airway obstruction and can be used to assess the symptom and disease severity in these patients [37]. So we choose the two indexes of pulmonary function to analyze the effectiveness of traditional Chinese exercise therapy which was different from the article [26]. Through a comprehensive observation of the FEV1 and Tiffenau Index indicators of pulmonary function, our meta-analysis results indicated that traditional Chinese exercises are superior to conventional therapy in a stable phase of patients with COPD. Meantime, in this article [27], it is also shown that Tai Chi can improve lung function for Patients with asthma and chronic obstructive pulmonary disease. Our findings support the findings of previous studies that investigated traditional Chinese exercises as an intervention for chronic cardiovascular diseases [38], cardiorespiratory fitness [39], and musculoskeletal diseases [40]. The reason for these positive effects may be that traditional Chinese exercise requires close coordination of breathing and movement, with the diaphragm lifting through slow, deep abdominal breathing; to achieve this coordination, consciously guided respiration is often used during the movements. This process significantly increases chest expansion and retraction, which leads to increased alveolar ventilation.

Decreases in daily life activities can cause harm to the body and mind and diminish the patient's quality of life. The most widely used method for the clinical assessment of quality of life of COPD patients is the CRDQ. This questionnaire not only reflects the patient's condition but also evaluates his or her psychological status, social activities, and daily life. Worldwide, there is good correlation between lung function and clinical symptoms, and race is not an influential factor. Subgroup analyses of dyspnea, fatigue, emotion and mastery in our study suggest that TCE resulted in improved quality of life compared with the control condition. Some research has demonstrated that TCE might improve quality of life in patients with other chronic diseases, such as heart failure, post-stroke impairments, and cancer [41–42]. Other studies have shown that outpatient pulmonary rehabilitation programs improve functional capacity and health-related quality of life [43–46]. Community-based respiratory rehabilitation and physical exercises also improve exercise tolerance and health-related quality of life [47]. We speculate that this effect may be related to social support, which may contribute to a sense of acceptance, and that TCE as a type of positive social interactions could offer companionship and friendly support. This, in turn, affects whole-body functioning and prevents unhealthy physiological behavioral responses, such as poor treatment adherence and social withdrawal, in patients with COPD. TCE was associated with improvements in exercise capacity, physical performance and quality of life of patients with COPD; thus, it offers an alternative form of therapy that does not require exercise equipment or a specific training venue. Therefore, it is possible to make effective exercise training more easily accessible for a large numbers of people with COPD, especially those with transportation problems or those living in remote and rural areas.

Rare adverse events were found in the studies included in our meta-analysis. The effects were mild and disappeared spontaneously after the first few sessions of training. In summary, traditional Chinese exercises seem to be well tolerated by patients with COPD.

Although we were different with article [22] which have included the articles only published in the English language, we excluded the low quality of the literature are all Chinese literatures. We believed that if they were included in the meta-analysis, may overestimate the results of the study and affect the effectiveness of the results which is different from several other articles [21–27]. Meantime, the low quality of the literature may have affected the evidence grades and clinical recommendations as mentioned in article [24]. Therefore, we only included literature with a high methodological quality, which may have made the results more conservative, robust and given them greater value as a clinical reference.

When meta-analyses are restricted to observational studies rather than experimental research, the results can be influenced by bias and confounding factors. Therefore, this study might have limitations in the following five areas. (1) The descriptions of the 10 studies regarding the randomization method, allocation concealment, blinding evaluation, loss outcomes and other aspects were not detailed and comprehensive; therefore, these included studies might exhibit moderate selection, implementation and measurement biases. (2) Because of the small number of included studies, it is impossible to further stratify the analysis according to age, gender, disease, intervention time, and intervention movement. (3) The intervention and follow-up times were not the same across all studies, which might also have influenced the authenticity of the pooled results.(4) The samples included in the study were not large, and we lacked large-scale RCTs, which might have affected the objectivity and reliability of the evaluation system.(5) Most of the studies lacked other objective outcome measurements, such as exacerbation rate, peripheral muscle strength, balance, survival and immune function, particularly at the molecular level.

## Conclusions

This meta-analysis of 10 RCTs showed that traditional Chinese exercises had rehabilitative effects on lung function, exercise tolerance and quality of life compared with conventional treatment in stable COPD patients.

## Supporting Information

S1 Appendix PRISMA 2009 checklist. (DOC)

S2 Appendix Reasons for excluded articles. (ZIP)
