# Supplementary material for: The Effects of Traditional Chinese Exercise in Patients with Chronic Obstructive Pulmonary Disease: A Meta-Analysis
Source: PLoS One. 2016 Sep 2;11(9):e0161564. doi: 10.1371/journal.pone.0161564 (PMC5010221; doi:10.1371/journal.pone.0161564)
Supplement: S2 Appendix — (ZIP) [file pone.0161564.s002.zip › S2 Appendix Reasons for excluded articles/Article1 .pdf]

Yeh et al. Trials 2014, 15:337
http://www.trialsjournal.com/content/15/1/337

TRIALS

STUDY PROTOCOL Open Access

Tai chi mind-body exercise in patients with COPD: study protocol for a randomized controlled trial

Gloria Y Yeh[1*], Peter M Wayne[4,5], Daniel Litrownik[1], David H Roberts[3], Roger B Davis[1] and Marilyn L Moy[2]

Abstract

Background: Chronic obstructive pulmonary disease (COPD) is a chronic, progressively debilitating condition that is prevalent in the US and worldwide. Patients suffer from progressive dyspnea and exercise intolerance. Physical exercise is beneficial, but conventional pulmonary rehabilitation programs are underutilized. There remains a need for novel interventions that improve symptoms, quality-of-life, and functional capacity. Tai chi is an increasingly popular mind-body exercise that includes physical exercise, breathing training, mindful awareness, and stress management–components that are essential to the self-management of COPD. There are, however, limited data on the effectiveness of tai chi as a therapeutic intervention in this population.

Methods/Design: The Primary Aims are to evaluate the efficacy, safety, and feasibility of a 12-week tai chi program for patients with COPD. We utilize a randomized controlled trial design, with participants assigned in a 2:1 ratio to either a group tai chi program (N = 63) or a time/attention-matched education control (N = 31). Our primary outcomes are COPD-specific quality-of-life and exercise capacity. Secondary outcomes include dyspnea, mood, functional status, self-efficacy, and lung function. Cardiopulmonary exercise testing is done in a subset of patients (N = 50). To explore optimal training duration, a subgroup of patients in tai chi are randomly assigned to complete an additional 12 weeks training (total 24 weeks) (Exploratory Aim 1). To explore the impact of a simplified seated intervention including only a subset of tai chi's training components, a third randomly assigned group (N = 31) receives a 12- week mind-body breathing program (N = 31) (Exploratory Aim 2).

Discussion: Results of the BEAM study (Breathing, Education, Awareness, Movement) will provide preliminary evidence regarding the value of tai chi for improving quality of life and exercise capacity in patients with COPD, including information regarding optimal duration. They will also inform the feasibility and potential benefit of an alternative mind-body breathing intervention, and provide insight regarding how isolated mind-body exercise components contribute to the overall effects of tai chi. Should the results be positive, tai chi and related mind-body practices may offer a novel exercise option that is potentially accessible to a large proportion of patients with COPD.

Trial registration: This trial is registered in Clinical Trials.gov, ID number NCT01551953. Date of Registration March 1 2012.

Keywords: Exercise, mind-body therapies, chronic obstructive pulmonary disease

* Correspondence: gyeh@hms.harvard.edu
[1]Division of General Medicine and Primary Care, Department of Medicine, Beth Israel Deaconess Medical Center, 1309 Beacon Street, Brookline, MA 02446, USA
Full list of author information is available at the end of the article

BioMed Central

© 2014 Yeh et al.; licensee BioMed Central Ltd. This is an Open Access article distributed under the terms of the Creative Commons Attribution License (http://creativecommons.org/licenses/by/4.0), which permits unrestricted use, distribution, and reproduction in any medium, provided the original work is properly credited. The Creative Commons Public Domain Dedication waiver (http://creativecommons.org/publicdomain/zero/1.0/) applies to the data made available in this article, unless otherwise stated.

Background

Chronic obstructive pulmonary disease (COPD), a progressive syndrome of airflow limitation [1], is a major cause of morbidity and mortality. In the US, it is the third most common cause of death, and is the only major disease among the top ten that continues to increase in prevalence [2]. In 2011, almost 13 million adults in the US were diagnosed with COPD, although close to 24 million have impaired lung function, indicating under-diagnosis [3,4].

Despite advances in pharmacologic and surgical therapy, patients suffer from dyspnea and have substantial limitations in daily activities. Airflow limitation puts the respiratory muscles at a mechanical disadvantage, increasing the work of breathing and worsening exercise tolerance. COPD also has systemic consequences with increased oxidative stress and systemic inflammation, contributing to skeletal muscle dysfunction and atrophy. In addition, persons with COPD are significantly less active than healthy persons [2] even at the earliest stages of disease [5,6]. In persons with COPD, decreased physical activity is associated with increased levels of systemic inflammation and increased risk of hospital admissions, acute exacerbations, and death, independent of lung function [7-10].

Moreover, anxiety and depression are prevalent among these patients [11], which can become additional barriers to participating in exercise and overall self–efficacy. Novel interventions that address complex, biopsychosocial issues of COPD and promote physical activity, could potentially improve symptoms, health-related quality-of-life (HRQL), functional capacity, and modify disease progression [2,12].

Complementary and integrative forms of exercise, such as tai chi, have gained popularity in the general population to promote health. Tai chi (*tai chi chuan* or *taijiquan*), is a multicomponent gentle, mind-body exercise that has its roots in ancient Chinese martial arts. It employs regimens of flowing circular movements that integrate balance, flexibility, strength, and breath training, along with multiple cognitive tools (for example, focused internal awareness, positive imagery) [13,14]. Tai chi's role in conventional medical management of chronic disease is poorly understood.

Studies have investigated tai chi exercise for a variety of medical diseases including cardiovascular conditions (for example, coronary artery disease [15], heart failure [7,16-18], hypertension [19,20]), fall prevention and neuromuscular control [21-26], pain and rheumatological conditions [27-29], and cognitive function and psychological wellbeing [30,31]. These studies support its potential health benefits and safety for chronic health conditions, and show promise as a novel, alternative, low-cost intervention for prevention and rehabilitation [32,33].

There have been few studies examining tai chi exercise specifically in patients with COPD. Recent meta-analyses suggest potential benefits for multiple outcomes including 6-minute walk distance, measures of dyspnea, spirometric indices, and quality of life. However, the evidence to date is preliminary and inconclusive due to small samples sizes and methodological flaws [34,35]. Moreover, the majority of these studies have been conducted in Asia, and generalizability of these results to ethnically diverse Western populations is unclear.

This clinical investigation, the BEAM study (Breathing, Education, Awareness, Movement), expands current knowledge of the potential efficacy of tai chi as an intervention for improving quality of life and exercise capacity in patients with COPD. First, this study is a rigorous investigation in a Western population utilizing a novel intervention protocol. Second, it provides an important evaluation of dosage and optimal duration of tai chi. Third, the study preliminarily investigates an alternative, simplified mind-body breathing (MBB) intervention that isolates breathing and meditative subcomponents of the tai chi intervention. Results of this study will both provide information about the relevance of these components in an overall tai chi program and also evaluate MBB as an alternative meditative intervention. Collectively, the BEAM study addresses key gaps in current COPD care by evaluating a potentially safe, modality of physical activity that is enjoyable, easily accessible, may lead to longer-term adherence, and become readily incorporated into COPD self-management plans.

Tai chi components relevant to COPD

Tai chi is a multicomponent intervention that integrates a number of training elements that are relevant to the treatment of patients with COPD [33,36]. Key elements included in our protocols include 1) physical exercise, 2) respiratory muscle training and breathing techniques, and 3) mindful awareness (Figure 1).

Physical exercise

The importance of exercise in the management of patients with COPD has been well studied, although there is ongoing debate regarding the optimal types, intensity, and duration of exercise [37-44]. Conventional exercise programs, both high- and low-intensity (such as walking, cycle ergometer, strength training), have been shown to improve exercise endurance, shortness of breath, and quality of life, even in patients with severe disease and poor exercise tolerance [45]. Most studies support that exercise leads to skeletal muscle adaptation, allowing more work to be performed.

Exercise may also desensitize patients to dyspnea. In addition, studies have suggested that unsupported upper extremity exercise (for example, against gravity) may offer additional benefits and effectively train patients in

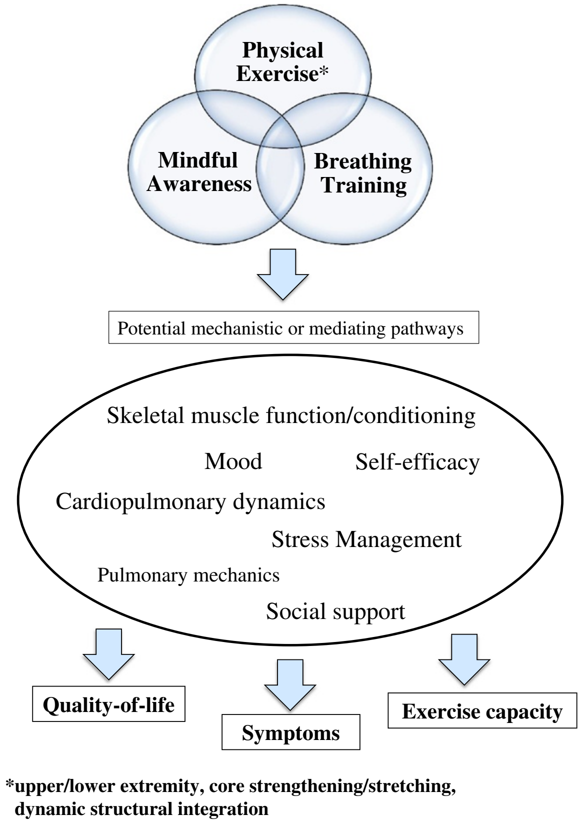

Figure 1 Conceptual model of tai chi and mind-body breathing and potential mechanistic pathways to improved patient-centered outcomes in chronic obstructive pulmonary disease (COPD). Physical activity, breathing training, and mindful awareness are the key elements of tai chi relevant to COPD. The potential mechanistic pathways through which tai chi may affect outcomes include skeletal muscle conditioning, cardiopulmonary dynamics, pulmonary mechanics, psychosocial factors (mood, self-efficacy, social support), and stress management. Favorable changes in these domains may then impact patient-centered outcomes of quality of life, symptoms, and exercise capacity. The mind-body breathing intervention contains similar key elements, although with less emphasis on physical activity (for example, less aerobic and no lower extremity or core strength training). The BEAM study includes the main patient-centered outcomes as well as secondary measures (quantitative or qualitative assessments) that inform each of the mechanistic pathways depicted in the center oval.

activities that mimic or are more similar to those required in daily living [39,46,47]. Although conventional pulmonary rehabilitation programs have been shown to be beneficial, only a small percentage of patients eligible for such programs actually participate and maintenance of physical activity is an ongoing challenge [48]. Alternative exercise options that are easily implemented, and that promote long-term adherence and self-efficacy, are much needed.

Tai chi provides mild aerobic activity, core strength training, and lower extremity and unsupported upper extremity training. The physical activity of tai chi is estimated to be mild-moderate aerobic exercise, at 1.6 to 4.6 metabolic equivalents (METs) and 50 to 74% maximal heart rate, depending on the age of the individual and the intensity of practice [49,50]. Previous literature suggests that it is safe in patients with chronic disease, including COPD, chronic heart failure, coronary artery disease (recovering from bypass surgery and myocardial infarction), as well as frailty, arthritis, and vestibular disease [51-53]. Studies also suggest that tai chi is accessible, enjoyable and has high rates of adherence; multiple comparative effectiveness studies have reported higher adherence to tai chi than the comparison group or exercise. Tai chi may provide a suitable range of exercise for those initiating physical activity or those transitioning to higher levels of activity, or as an adjunct to other forms of conventional activity.

Respiratory muscle training and breathing techniques

In COPD, respiratory muscle weakness and inefficiency contributes to breathlessness, and exercise impairment. Respiratory muscle training selectively works the inspiratory muscles to perform against loads, thereby increasing strength, endurance, and efficiency. Studies in patients with various respiratory disease, including COPD, have reported improvements in inspiratory muscle function (maximal inspiratory pressure), exercise performance (walk distance), and dyspnea related to daily activities [54-56].

Similarly, breathing retraining in COPD aims to teach patients to breathe more efficiently, replacing rapid shallow breathing patterns that may worsen gas exchange with slower breathing patterns that improve chest wall mechanics, allow more complete expiration, and decrease air trapping. Conventional breathing retraining, such as diaphragmatic and pursed-lip breathing, may improve symptoms, increase tidal volume and total ventilation, decrease respiratory rate, and improve gas exchange [57-59]. In addition, slow, deep breathing can also have beneficial effects on the autonomic nervous system which is increasingly recognized as highly relevant in COPD [60]. Studies in COPD patients have suggested changes in sympathetic/parasympathetic balance in indices such as heart rate variability [61].

Efficient, intentional and mindful breathing is a key element of tai chi. The interventions employed in the BEAM study include elements of respiratory muscle and breathing retraining that similarly aim to increase muscle strength and endurance, decrease mechanical loads such as chest wall stiffness, and deepen and slow the respiratory rate, thereby increasing gas exchange efficiency. The MBB is taught and integrated within a broader context of relaxation, focused self-awareness, and imagery (see below), which may easily be adopted by patients and facilitate translation into usual breathing patterns during activities of daily life.

Mindful awareness

A fundamental component of tai chi is the deliberate attention to bodily sensation, movement, breath, and emotion, which fosters acute self-awareness, both physically and emotionally. Participants learn, for example, to discriminate areas with or without strain or tension, stronger or weaker regions, movements that feel graceful or fearful, or aspects of breathing that feel labored or unconstrained. This awareness may in turn complement other tai chi components that foster improved function (for example, improved posture, relaxation). Recent studies support that mindfulness training can impact interoceptive awareness of key COPD symptoms (such as dyspnea and cough) which may lead to better symptom management [62-64]. Inner awareness of moment-to-moment sensations also helps develop focused attention, providing a tool to manage distracting thoughts [65].

We hypothesize that increased awareness of postures and breathing, and the psychophysiology of when dyspnea starts, will allow participants to better anticipate and manage their symptoms, decreasing the risk of worsening dyspnea and precipitating further breathing difficulty and anxiety. This heightened mindfulness and self-awareness of breathing, body shape, and emotion may be important in facilitating change in pulmonary patients who have developed maladaptive physical and psychological patterns due to chronic dyspnea over time, and lead to better symptom management [63]. In addition, it is increasingly recognized that patients with COPD are at high risk of developing symptoms of anxiety and depression [66]. Anxiety over dyspnea-producing activities is common and may promote maladaptive sedentary lifestyles [11]. Collectively, the mind-body approach of tai chi inherently addresses stress management which is an important component of COPD self-management.

Methods/Design

The BEAM study is a pilot randomized controlled trial investigating tai chi exercise and MBB as an adjunct to standard care in patients with COPD. We will enroll a total of 125 participants, with participants randomized in a 2:1 ratio to either a 12-week tai chi program (n = 63) or a time- and attention-matched education control (n = 31).

Aims/hypotheses

The primary aims of this study are to evaluate the 1) efficacy and 2) safety and feasibility of a 12-week tai chi mind-body exercise for patients with COPD. To explore optimal training durations of tai chi, a subgroup of patients assigned to the tai chi program are randomly assigned to complete an additional 12 weeks of training (total 24 weeks) (Exploratory Aim 1). Additionally, to explore the impact of an alternative simplified seated intervention that includes a subset of tai chi training components, a third randomly assigned group (n = 31) receives a 12-week MBB program (n = 31) (Exploratory Aim 2) (Figure 2).

Our primary efficacy outcomes are COPD-specific quality-of-life (chronic respiratory questionnaire (CRQ)) and exercise capacity (6-minute-walk test). Secondary outcomes examining further psychosocial and physiological effects of tai chi include symptoms of dyspnea, fatigue, mood, functional status, strength and flexibility, self-efficacy, social support, and pulmonary function. Cardiopulmonary exercise testing is also done in a subset of patients (n = 50). Safety and feasibility will be assessed by successful recruitment and randomization, adherence to the protocol and attendance at classes (at least 70%), and demonstrated safety through systematic adverse events (AE) monitoring and reporting.

We hypothesize that there will be improved patient-centered outcomes (quality of life and exercise capacity) in the tai chi group as compared to education control and that the study will be feasible and safe in COPD. Those who receive continued tai chi in phase 2 will have increased 24-week adherence and improved outcomes. There may be similar benefit in the MBB group in symptoms of dyspnea and psychosocial measures, but the tai chi intervention will have trends towards more benefit on exercise capacity, cardiopulmonary function, strength, and flexibility.

Study population

Participants are being identified from the primary care and pulmonary clinics at two primary institutions: Beth Israel Deaconess Medical Center (BIDMC) and VA Boston Healthcare System. Additional recruitment sites include the pulmonary clinics at Brigham and Women's Hospital and Boston Medical Center. Recruitment strategies include direct referral from physicians, and identification of eligible patients through hospital databases, and mailings. All recruitment and data collection procedures follow the Health Information Privacy and Accountability Act (HIPAA) guidelines to maintain patient confidentiality, and study procedures have been approved by each institution's investigational review board. A list of the institutions and ethical boards providing approval is provided in Additional file 1.

Participants are eligible for the study if they: 1) have moderate-severe COPD as defined by global obstructive lung disease (GOLD) stage 2, 3, or 4, with symptoms of dyspnea (either forced expiratory volume in one second (FEV1) ≤80% and FEV1/forced vital capacity (FEV1/FVC) <0.70, or evidence of emphysema on computed tomography (CT); and 2) are aged ≥40 years.

Exclusion criteria are: 1) respiratory failure or GOLD stage 4 and inability to safely perform a 6-minute walk test or otherwise exercise safely as deemed by a

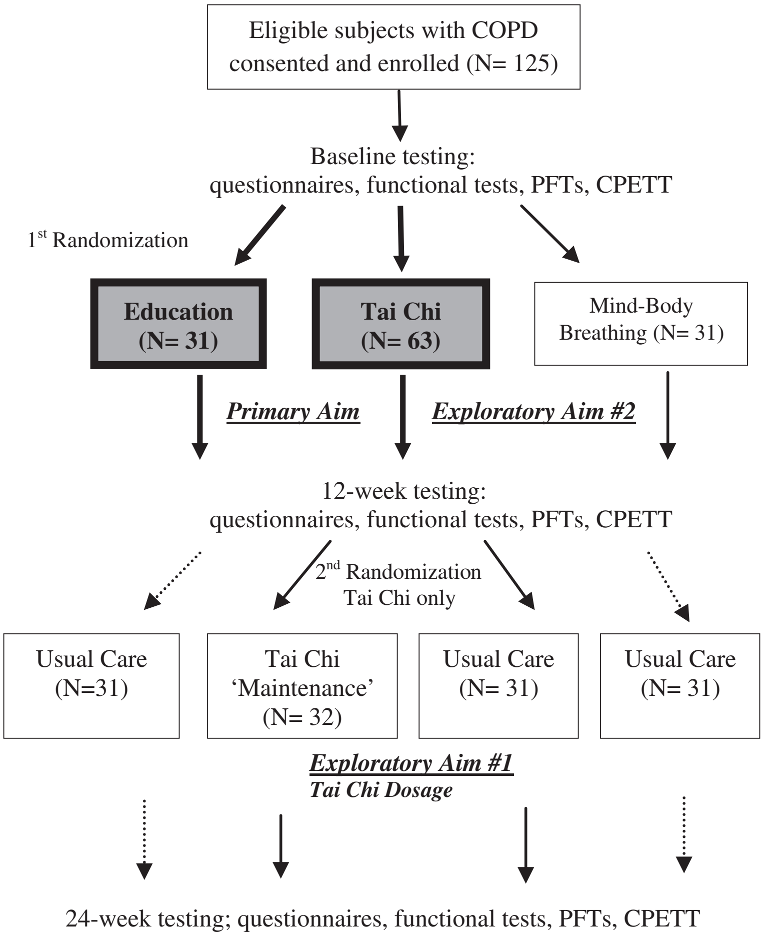

Figure 2 The BEAM study design. The primary aims of this randomized controlled trial are to evaluate the efficacy, safety and feasibility of a 12-week tai chi exercise program as compared to a time- and attention-matched education control. At 12 weeks, subjects in tai chi undergo a second randomization to either continue with tai chi for an additional 12 weeks (total 24 weeks), or to receive usual care. (Exploratory Aim 1: Tai Chi Dosage). To explore the impact of a simplified seated mind-body breathing intervention, a third randomly assigned group receives a 12-week mind-body breathing program (Exploratory Aim 2). After the initial 12 weeks of both education and mind-body breathing, subjects receive usual care for the following 12 weeks. All participants undergo testing at baseline, 12 weeks, and 24 weeks.

physician; 2) COPD exacerbation requiring steroids, antibiotics, an emergency room (ER) visit or hospitalization within the past 2 weeks unless the physician deems the subject to be at clinical baseline; 3) planned thoracic surgery within the next 3 months; 4) hypoxemia on the walk test or cardiopulmonary exercise test (oxygen saturation <88% on supplemental oxygen); 5) inability to ambulate due to vascular or other neuromuscular conditions that would preclude a walk test; 6) clinical signs of unstable cardiovascular disease (that is, chest pain on the walk test or electrocardiogram changes on the cardiopulmonary exercise test); 7) severe cognitive dysfunction (mini-mental status exam score ≤24); 8) non-English speaking; 9) current active participation in a pulmonary rehabilitation program or current regular practice of tai chi; and 10) physician diagnosis of unstable/untreated clinical depression.

Informed consent

Written informed consent is obtained by the research assistant at the initial visit. Patients are asked to participate in a study examining three types of intervention involving exercise and/or education. In order to assess credibility of the three groups and clinical equipoise from the patient's perspective, we employ an expectancy and treatment credibility assessment from Borkovec and Nau. This instrument provides a measure of how credible patients perceive a treatment to be, and has been used in prior trials to assess blinding [67].

Randomization and allocation concealment

Randomization occurs after baseline testing. Participants are randomized to one of three group classes: tai chi, MBB, or education, in a 2:1:1 ratio. Group assignments are generated by a permuted blocks method with randomly

varying block size, and sealed in numbered, opaque envelopes. Intervention classes begin within 3 weeks of baseline testing. At the end of phase 1, participants in the tai chi group undergo a second randomization to either continue tai chi class once weekly, or to resume usual care. Participants are informed at baseline of the two randomization processes and that the intervention may be either 3 or 6 months long.

Interventions

Tai chi, MBB, and education classes are conducted twice weekly for one hour each for the first 12 weeks. In the second 12 weeks of the study, participants in the tai chi class may be assigned to continue tai chi classes once weekly for an additional 12 weeks. Instructors who administer the tai chi and MBB interventions are senior tai chi students and certified graduates of a 2.5-year instructor training program that includes training in meditative breathing. The average training experience of the four study instructors is 19 years.

Importantly, the tai chi instructors also administer the education class to minimize the variability with respect to instructor type, personality, and training. Chairs are provided for seated warm-up, breathing exercises and resting, as well as for stability as needed when performing standing tai chi exercises. Subjects in tai chi and MBB groups receive a 45-minute video as well as a separate audio file of the exercises taught in class, and are given detailed instruction about specific exercises, and encouraged to practice outside of class three times a week (for at least 30 minutes).

Tai chi intervention

The 12-week tai chi intervention has been specifically designed for an older, physically limited population with COPD. In brief, this protocol was modified from a similar intervention that was developed in prior studies investigating tai chi for chronic heart failure [7,68] and in a pilot study of COPD patients [52].

We convened an expert panel with representation from pulmonary medicine, exercise physiology, mind-body medicine, tai chi, and clinical trial methodology. We refined the intervention protocol using an iterative modified Delphi process (details to be reported elsewhere). In comparison to our intervention for persons with heart failure, the current tai chi protocol further emphasized the integration of multiple mindful breathing techniques as well as the addition of two warm-up exercises to improve awareness and flexibility of the spine, rib cage and upper torso.

The intervention emphasizes essential tai chi movements that are both easily comprehensible and can be done repetitively in a flowing manner. The five tai chi movements - ‘raising the power’, ‘withdraw and push’, ‘grasp the sparrow’s tail’, ‘brush knee twist step’, and ‘cloud hands’ - are based on the traditional Cheng Man-Ch’ing’s Yang-style short form [13]. In addition to these five core movements, the intervention includes a complementary set of traditional tai chi warm-up exercises, including specific MBB techniques. These warm-ups focus on loosening the physical body, providing moderate aerobic activity, incorporating mindfulness and imagery into movement, promoting overall relaxation of body and mind, and generating awareness and efficiency of breathing.

The MBB techniques included within the tai chi protocol are described below. All components of the MBB intervention are included in the tai chi intervention. Within the tai chi protocol, breathing components are taught both in a seated position as in the MBB class, but also integrated into moving tai chi and warm-up exercises. We conclude each session with a brief cool-down exercise of seated self-massage of the face, abdomen, flanks, and mid-back.

Education control

The content of this class is based on information from the American Thoracic Society, American College Chest Physicians, and the *Global Obstructive Lung Disease Patient Guide* [69-74]. Educational modules include: anatomy of the lungs, COPD, managing COPD symptoms, smoking cessation, diagnostic tests, understanding COPD meds, managing acute exacerbations, managing stress, exercise, nutrition, sleep, mental health, oxygen therapy, surgical options, pulmonary rehabilitation, and advance care planning. Time is spent with both didactic as well as informal group discussion moderated by the instructor. The instructors have explicit instructions to moderate discussions and not make any recommendations regarding an individual’s treatment.

MBB intervention

The MBB intervention is comprised of the breathing techniques of the tai chi exercise program; however, all techniques are performed in a seated position. This intervention removes some elements of physical activity (no aerobic activity, no lower extremity strengthening and stretching, less core strengthening), while retaining breathing and mindful awareness.

The overall goals of the breathing intervention are to increase the efficiency of gas exchange, enhance awareness/mindfulness of the mechanics of breathing, and promote relaxation of both physical body and mind. Four traditional inter-related techniques are taught; detailed descriptions of these can be found in traditional and modern tai chi and *qigong* publications [75-78]. 1) *renewing the body with breath* is a simple relaxation technique that employs the breath and imagery as a

focusing tool to release physical and emotional tensions; 2) *mindful breathing* cultivates inner awareness of the breath as air and its consequent internal pneumatic and hydraulic pressures travel from the nostrils to the lower abdomen, and back out; 3) *ocean breathing* emphasizes diaphragmatic breathing and expansion and contraction of the lower abdomen; and 4) *balloon breathing* emphasizes effortless inhalations and a prolonged exhalation cycle relative to inhalation.

Each of the techniques was chosen to emphasize different aspects of MBB relevant to COPD rehabilitation. The expectation is that over time these methods will become integrated into usual breathing patterns during daily activities. Subjects in class are explicitly taught to incorporate the techniques into simple activities of daily living (for example, while reading or talking on the phone). The protocol also begins with the same series of seated warm-up exercises that are employed in the tai chi intervention that particularly target awareness and flexibility of the upper torso and rib cage. Subjects who complete all study requirements have the option of joining a class of their choice (one of the two groups to which they were not randomized). These subjects do not undergo additional testing but are monitored for AEs.

Primary measures

All research staff performing outcome testing are blinded to participants' intervention allocations. Table 1 details the schedule of evaluations.

Six-minute walk test

This is a standardized assessment that measures the maximum distance walked in 6 minutes [79]. Subjects are read standardized, scripted instructions and informed when there are 3 minutes and 1 minute before the end of the test. Subjects are allowed to stop as often as they need and to use supplemental oxygen if usually prescribed for activity. The 6-minute walk test has been shown to be an independent correlate of COPD prognosis and survival [80,81].

Disease-specific HRQL measure

The main HRQL measure is the disease-specific chronic respiratory disease questionnaire (CRQ). This validated instrument is one of the most commonly employed health status measures for COPD. It consists of 20 items covering four domains - dyspnea, fatigue, emotional function and mastery. Items are scaled on a 7-point modified Likert scale, with higher scores indicating better HRQL [82]. We use the self-administered, standardized

Table 1 Schedule of evaluations

Outcome	Measurement/instrument	Month		
		0	12	24
Physical functioning				
Exercise capacity	**6-minute walk test (6 MW)***	X	X	X
	Bicycle cardiopulmonary exercise test**			
Strength/flexibility	Chair sit and reach	X	X	X
	Chair stand			
Physical function	PROMIS physical function	X	X	X
Health-related quality-of-life and symptoms (HRQL)				
COPD-specific HRQL	**Chronic respiratory disease questionnaire (CRQ)***	X	X	X
Dyspnea	UCSD shortness of breath questionnaire;	X	X	X
	MMRC dyspnea scale			
Fatigue	PROMIS fatigue	X	X	X
Psychosocial functioning and support				
Self-efficacy	COPD self-efficacy scale	X	X	X
Mood	Center for epidemiologic studies-depression scale (CES-D)	X	X	X
Stress	Perceived stress scale	X	X	X
Perceived social support	Multidimensional scale of perceived social support	X	X	X
Pulmonary function				
Spirometry and lung volumes	Standard pulmonary function tests	X	X	X

*Primary measures. **Cardiopulmonary exercise test (n = 50) will be done in a subset of patients. PROMIS, Patient-Reported Outcome Measurement Information System; COPD, chronic obstructive pulmonary disease; UCSD, University of California, San Diego; MMRC, Modified Medical Research Council.

version. Our main outcome is a total CRQ score calculated by the sum of individual responses divided by the number answered (mean response).

Secondary measures

Cardiopulmonary exercise test

In a random subset (n = 50 total), participants perform a symptom-limited exercise test using a bicycle ramp protocol to determine peak oxygen uptake (VO_2) and exercise endurance. Testing is done on an electronically calibrated upright bicycle, with expired gas analysis under continuous electrocardiographic monitoring. Breathlessness and leg fatigue are measured using the Borg scale of 1 to 10 during the test. Breath-by-breath respiratory gas analysis is performed using a SensorMedics (Yorba Linda, CA, USA) metabolic cart. Peak values are averaged from the final 20 seconds of the test. Peak VO_2 has been shown to correlate with cardiac output and skeletal muscle blood flow and predict mortality in COPD [83].

Strength and flexibility

The chair stand test assesses lower body strength and endurance. Subjects are instructed to rise to a full standing position and then return to a seated position with arms folded across their bodies as many times as possible within 30 seconds. This validated test has been used in numerous populations with age and gender specific normative data available [84]. The chair sit and reach assesses lower body flexibility, primarily the hamstring, and is a modification of the original sit and reach, developed for use in older or deconditioned populations to decrease risk of injury in those with back pain or limited range of motion [85].

Dyspnea

The University of California, San Diego (UCSD) shortness of breath questionnaire is a 24-item self-administered instrument that assesses the degree to which patients feel short of breath while performing 21 different activities of daily living. Respondents rate symptoms on a 6-point scale from not at all to maximally or unable to do because of breathlessness [86]. The Modified Medical Research Council (MMRC) dyspnea scale is a 4-point scale assessing dyspnea severity, with a score of 4 indicating that the patient is too breathless to leave the house or becomes breathless when dressing or undressing. This scale predicts the likelihood of survival among patients with COPD and correlates well with other scales and health status scores [87]. This scale is one of the four components of the body mass: airflow obstruction: dyspnea: exercise capacity (BODE) index, a validated multidimensional grading system for COPD that has been shown to be a better predictor of mortality than FEV_1 alone [88].

Self-efficacy

The COPD self-efficacy scale (CSES) was developed to identify situations during which patients with COPD lack confidence in their ability to manage or to avoid breathing difficulties. The situations include times of negative affect (*when I feel down or depressed*), intense emotions, physical exertion, at-risk behaviors (*when I overeat*), or adverse weather/environmental conditions. Patients rate the level of confidence they feel, ranging from not confident at all to very confident, in managing or avoiding breathing difficulties during 24 different situations. The CSES has high internal consistency ($r = 0.95$) and test-retest reliability ($r = 0.77$) [89].

Emotional status/mood

The Center of Epidemiology Studies-depression scale (CES-D) is a general measure of psychological impairment, primarily depressive symptoms, that has been used extensively in epidemiology studies [90]. It is a validated instrument consisting of 20 items, including feelings of depression, worthlessness, loneliness, energy level and fear. Participants are asked to report how often they experienced the symptom during the past week using a 4-point ordinal scale (rarely or none of the time; some or little of the time (1 to 2 days); occasionally or moderate amount of the time (3 to 4 days); most or all of the time (5 to 7 days). A score <15 indicates no depression. The CES-D has high internal consistency ($r = 0.90$) and a test-retest reliability of 0.51 [91,92].

Physical function and fatigue

We use the PROMIS Physical Function-Short Form 10a questionnaire to track patients' physical functioning, and the PROMIS Fatigue Short Form 7a to track patients' fatigue. The Patient-Reported Outcome Measurement Information System (PROMIS®) instruments funded by the National Institutes of Health are based on modern measurement theory and include the application of mixed methods approaches for instrument development. Both forms are short validated instrument with seven to ten items, each with five response options on an ordinal scale (not at all, a little bit, somewhat, quite a bit, very much) [93,94].

Perceived stress and social support

The perceived stress scale is a measure of the degree to which situations in one's life are appraised as stressful. We will use the 10-item version of this instrument which we have used in our prior tai chi trials and which has been shown to have good reliability and validity [95]. The multidimensional scale of perceived social support is a validated instrument that will be used to assess the degree of perceived social support provided in each

group. It consists of 12 items covering subscale areas of family, friends, and significant others [96].

Pulmonary function tests

Spirometry and lung volume measurements will be performed using rolling-seal volume displacement spirometers (Collins CPL Raptor, Louisville, CO, USA), following American Thoracic Society standards for quality and reproducibility [97]. Lung volumes are performed via plethysmography. Lung volumes, such as total lung capacity (TLC) and functional residual capacity, add information about hyperinflation from COPD that may negatively affect respiratory muscle function. The inspiratory-to-total lung capacity ratio (IC/TLC) may be a particularly useful index of resting airflow limitation and lung hyperinflation that correlates with exercise capacity [98].

Other data collection

Physical activity

To track participants' level of physical activity outside of exercise classes, we use the Community Health Activities Model Program for Seniors (CHAMPS) physical activity questionnaire for older adults [99]. CHAMPS is a 41-item validated instrument in the elderly, which covers physical activity from several domains, including leisure, household, and occupational. Weekly frequency and total time spent allows estimation of caloric expenditure. In addition, we use the Moy physical activity checklist specifically to measure activity in patients with COPD [100]. A higher number of daily checklist activities performed is associated with better indices of COPD health, including higher FEV_1 and lower BODE index.

Qualitative interview

We also perform semi-structured qualitative exit interviews with participants in all groups to further explore areas not captured in our standardized quantitative instruments. Questions probe participants' experiences with the program overall; changes in physical, mental, or social function specific to the intervention; changes in illness perception; experiences with practicing the intervention; and expectations and beliefs about mind-body therapies. These open-ended questions may yield additional insights into understanding the mechanism of a mind-body exercise program. Each interview session is audio recorded, then transcribed verbatim.

Adherence

Adherence is defined as greater or equal to 70% of intervention classes attended. During phase 1 in the first 12 weeks, compliance with home practice in the tai chi and MBB arms is tracked through home practice logs that are completed at each class. During phase 2 in the second 12 weeks, those not randomized to continued classes are queried about home practice once a month by phone.

Safety and adverse event monitoring

Interview data are used to document patients' use of health care during the study period. Medical record review is conducted for details of hospitalizations for AE reporting. At each testing visit at baseline, 12 and 24 weeks, we ask specifically about medical symptoms in the past 12 weeks such as muscle strain, fainting/loss of consciousness, dizziness, worsening shortness of breath or COPD exacerbation, worsening fatigue, falls, palpitations, psychological stress, or other AEs. For any symptom ascertained, we further document the patient's perception of relatedness to the intervention or testing, severity, any required change in medications, and any further medical care, hospitalization or ER visit. For hospitalizations and ER visits, we further ascertain discharge diagnosis and results of major cardiopulmonary tests or procedures performed. During the intervention, occurrences of adverse events are also queried by instructors and captured in the attendance log, which subjects complete during each class. If a potential AE is reported, study staff contact the participant for further details to determine if a reportable AE has occurred and if so, document the type, whether expected or not, relatedness to the study, and severity. Serious AEs are defined according to our institutional review board's policy to be an event that is life-threatening, requires hospitalization, results in persistent or significant disability/incapacity or death. Incidents are reported to the IRB, our sponsor, and our Data Safety and Monitoring Board (DSMB) as appropriate.

Statistical analysis

Sample size and power

This study is powered on our pre-specified primary outcomes of overall CRQ (chronic respiratory questionnaire) score and 6-minute walk-test distance. Our effect estimates and standard deviations are based on pilot data of 12-weeks of tai chi versus usual care in patients with COPD [52]. Enrollment of 125 participants will provide approximately 93% power to detect differences between groups of approximately 50 meters change in the 6-minute walk test and 0.9 point change in total CRQ score (mean response value). Our primary comparison will be between the tai chi and education groups, from baseline to 12 weeks in phase 1. It is widely accepted that a change in the 6-minute walk test of 54 meters is clinically significant [81,101-104]. For the CRQ, Redelmeir *et al*. reported that a 0.5 point change is clinically meaningful [104]. Our study has over 96% power to detect a difference of 54 meters and 99% power

to detect a difference of 68 meters (the difference observed in our pilot). These calculations allow for a 5% loss of statistical efficiency of the nonparametric test compared to a *t*-test (for the 6-minute walk), 10% dropout rate, and a conservative Bonferroni correction for multiple outcomes.

For our exploratory dosage aim, we expect to have about 82% power to detect differences between groups of a 50-meter change in 6-minute walk and 0.9 point change in total CRQ. This sample size of 21 per group (12 weeks versus 24 weeks of tai chi) accounts for a 5% loss of statistical efficiency. We expect to have limited power for our second exploratory aim comparing tai chi to the MBB group.

Prior to analyzing the outcomes, we will examine baseline demographic, functional, and clinical variables as well as known prognostic factors (such as baseline GOLD stage and baseline peak VO_2) by randomized group. Our primary analyses will examine changes between baseline and 12 weeks.

Primary aims

Efficacy Changes in total scores on the CRQ will be analyzed using analysis of covariance (ANCOVA), comparing the change from baseline to 12 weeks among the two treatment groups tai chi versus education, adjusted for the baseline value of the outcome (and, if necessary, baseline characteristics found poorly balanced between groups). For the 6-minute walk, the change from baseline to 12 weeks is known to be skewed, so we will use a nonparametric approach and use a Wilcoxon rank sum test to compare the two treatment groups at $\alpha = 0.05$ (without the Bonferroni correction, given the pilot feasibility nature of the study). As complements to these main approaches, we may use bootstrap analysis to evaluate confidence intervals and the cumulative distribution function with evaluation of the area under the curve for each study arm to show the distribution of patients who improve.

Safety/feasibility Study feasibility will be established if we can report that a reasonable proportion of eligible patients are willing to participate (>10%), that we can recruit participants at a reasonable rate (approximately 5 patients per month), and that patients once enrolled will adhere to our study protocol (with at least 70% attendance). Safety will be evaluated through descriptive statistics of AEs and frequencies will be reported per type according to grade and severity per arm. The intervention will be considered safe if there are no serious AEs related to the intervention.

Secondary aims

The basic analytic approach for secondary efficacy measures will be parallel to the approach for the primary measures, comparing change in outcome from baseline to 12 weeks among the two treatment groups. However, for these secondary measures, we do not anticipate using an adjustment for multiple outcomes. As secondary analyses, we also plan to analyze the change in each of the four CRQ subdomains (dyspnea, fatigue, emotion, mastery) using the same methods.

In addition, we will use linear regression models using primary study outcomes (change in 6-minute walk and CRQ score) as dependent variables and scores on psychosocial assessments and functional tests as potential mediators to examine whether clinical outcome is associated with these psychosocial or physiological parameters. Separate exploratory models will be developed for each primary outcome, providing insight into potential mechanisms of effect.

Qualitative exit interviews will be professionally transcribed from audio recordings and coded using an inductive approach informed by grounded theory methods. We will identify passages in the transcripts that represent common themes or content categories relating to positive, negative, and neutral aspects of patients' experiences with tai chi, MBB, and education control. Data will be analyzed and presented descriptively according to content categories (as above) [105-108]. We will also examine a per-protocol analysis of our primary outcomes for aim 1, where non-adherers will be excluded (secondary analysis). Non-adherence will be defined as attendance at less than 70% of classes.

Exploratory aim 1 (dosage) With respect to examining dosage of tai chi, we will compare the change in all outcomes between the two tai chi groups (12-week versus extended 24-week intervention) using ANCOVA and the Wilcoxon rank sum test as appropriate, similar to our primary aims. We will further assess adherence to the continued classes from 12 to 24 weeks. We will also analyze home practice hours (in both groups), and total practice hours (class plus home practice in the 24-week class group) and compare groups using the Wilcoxon rank sum test. Clinical data obtained through patient interview will be analyzed using descriptive statistics.

Particularly in the intervention groups, we will describe patient attendance at classes and compliance with home practice, and examine whether compliance or dosage is associated with clinical outcome.

Exploratory aim 2 (MBB) We will obtain the point estimates for the change in outcomes, that is, mean change in CRQ and median change in the 6-minute walk between baseline and 12 weeks in the MBB group. We expect these means/medians to be intermediate between changes in the tai chi group and changes in the education group. Even though we are not powered to detect

differences between groups, we will conduct exploratory analyses to gather preliminary estimates of effect. We will use ANCOVA and Wilcoxon rank sum tests, similar to the first aim, to compare change in the CRQ and the 6-minute walk, between the MBB group and education, and between the MBB group and tai chi. We will establish overall feasibility and adherence of the MBB group by assessing willingness to participate and attendance at classes.

Summary/Discussion

Results of this innovative study will establish feasibility and provide preliminary evidence on the efficacy of tai chi exercise to improve quality of life and exercise capacity in patients with COPD. In addition, we will capture information on potential physiological and psychosocial mechanistic pathways. The development and exploration of a seated intervention that emphasizes meditative breathing provides further insight into mechanisms of tai chi by isolating relevant elements of the larger, multi-component intervention. Finally, the extension of intervention duration from 12 to 24 weeks will help to inform issues of optimal dosage for this population.

Trial status

This study is currently ongoing with active recruitment.

Additional file

Additional file 1: List of approving Institutional Review Boards.

Abbreviations

AE: adverse event; ANCOVA: analysis of covariance; BEAM: breathing: education: awareness: movement; BIDMC: Beth Israel Deaconess Medical Center; BODE: body mass: airflow obstruction: dyspnea: exercise capacity; CES-D: Center of Epidemiology Studies-depression scale;
CHAMPS: Community Health Activities Model Program for Seniors physical activity questionnaire for older adults; COPD: chronic obstructive pulmonary disease; CRQ: chronic respiratory disease questionnaire; CSES: chronic obstructive pulmonary disease self-efficacy scale; DSMB: Data Safety and Monitoring Board; ER: emergency room; FEV1/FVC: forced expiratory volume in one second/forced vital capacity; GOLD: global Obstructive Lung Disease; HIPAA: Health Information Privacy and Accountability Act; HRQL: health-related quality-of-life; IC/TLC: inspiratory-to-total lung capacity ratio; IRB: Institutional Review Board; MBB: mind-body breathing; MET: metabolic equivalent; MMRC: Modified Medical Research Council; PROMIS®: Patient-Reported Outcome Measurement Information System; TLC: total lung capacity; VO2: peak oxygen uptake.

Competing interests

Peter Wayne is the founder and sole owner of the Tree of Life Tai Chi Center. Peter Wayne's interests were reviewed and are managed by the Brigham and Women's Hospital and Partners HealthCare in accordance with their conflict of interest policy.

Authors' contributions

GY conceived of the study, led in its design and coordination and drafted the manuscript. PW participated in study design and coordination and assisted in drafting the manuscript. DL participated in study coordination and assisted in editing the manuscript. DR participated in the design of the study and assisted in coordination. RD participated in design of the study and development of the statistical analysis plan. MM participated in design and coordination of the study and assisted in drafting the manuscript. All authors read and approved the final manuscript.

Acknowledgements

We would like to thank Amy Brown, Elizabeth Kacel, Caroline Chan, Cecilia Griggs, Danielle Berkowitz, Morgann Young, Danielle Mullin, Diana Homsy, Ana Kantorowski, and Merilee Teylan for their assistance in implementing this study. This study was supported by an award from the National Center for Complementary and Alternative Medicine (NCCAM) at the National Institutes of Health (NIH) (R01AT005436). Its contents are solely the responsibility of the authors and do not necessarily represent the official views of the NCCAM or the NIH.

Author details

[1]Division of General Medicine and Primary Care, Department of Medicine, Beth Israel Deaconess Medical Center, 1309 Beacon Street, Brookline, MA 02446, USA. [2]Pulmonary and Critical Care Section, Department of Medicine, Veterans Administration Boston Healthcare System, 1400 VFW Parkway, Boston, MA 02132, USA. [3]Division of Pulmonary, Sleep and Critical Care Medicine, Beth Israel Deaconess Medical Center, 330 Brookline Ave, Boston, MA 02215, USA. [4]Osher Center for Integrative Medicine, Harvard Medical School, 900 Commonwealth Ave, Boston, MA 02446, USA. [5]Division of Preventive Medicine, Brigham and Women's Hospital, Boston, MA, USA.

Received: 10 June 2014 Accepted: 22 July 2014
Published: 28 August 2014

References

1. Sutherland ER, Cherniack RM: **Management of chronic obstructive pulmonary disease.** *N Engl J Med* 2004, **350:**2689–2697.
2. Mannino DM, Homa DM, Akinbami LJ, Ford ES, Redd SC: **Chronic obstructive pulmonary disease surveillance–United States, 1971–2000.** *MMWR Surveill Summ* 2002, **51:**1–16.
3. Centers for Disease Control and Prevention. National Center for Health Statistics: **National Health Interview Survey Raw Data, Analysis performed by the American Lung Association Research and Health Education Division using SPSS and SUDAAN software.** 2011.
4. Centers for Disease Control and Prevention: **Chronic Obstrcutive Pulmonary Disease Surveillance - United States, 1971–2000.** *Morb Mortal Wkly Rep* 2002, **51:**1–16.
5. Decramer M, Rennard S, Troosters T, Mapel DW, Giardino N, Mannino D, Wouters E, Sethi S, Cooper CB: **COPD as a lung disease with systemic consequences–clinical impact, mechanisms, and potential for early intervention.** *COPD* 2008, **5:**235–256.
6. Moy ML, Danilack VA, Weston NA, Garshick E: **Daily step counts in a US cohort with COPD.** *Respir Med* 2012, **106:**962–969.
7. Yeh GY, Wood MJ, Lorell BH, Stevenson LW, Eisenberg DM, Wayne PM, Goldberger AL, Davis RB, Phillips RS: **Effects of tai chi mind-body movement therapy on functional status and exercise capacity in patients with chronic heart failure: a randomized controlled trial.** *Am J Med* 2004, **117:**541–548.
8. Moy ML, Teylan M, Weston NA, Gagnon DR, Danilack VA, Garshick E: **Daily step count is associated with plasma C-reactive protein and IL-6 in a US cohort with COPD.** *Chest* 2014, **145:**542–550.
9. Moy ML, Teylan M, Weston NA, Gagnon DR, Garshick E: **Daily step count predicts acute exacerbations in a US cohort with COPD.** *PLoS One* 2013, **8:**e60400.
10. Moy ML, Teylan M, Danilack VA, Gagnon DR, Garshick E: **An index of daily step count and systemic inflammation predicts clinical outcomes in chronic obstructive pulmonary disease.** *Ann Am Thorac Soc* 2014, **11:**149–157.
11. Hill K, Geist R, Goldstein RS, Lacasse Y: **Anxiety and depression in end-stage COPD.** *Eur Respir J* 2008, **31:**667–677.
12. Crisafulli E, Costi S, Luppi F, Cirelli G, Cilione C, Coletti O, Fabbri LM, Clini EM: **Role of comorbidities in a cohort of patients with COPD undergoing pulmonary rehabilitation.** *Thorax* 2008, **63:**487–492.
13. Cheng M: *Master Cheng's Thirteen Chapters on T'ai Chi Chuan.* New York: Sweet Chi Press; 1982.
14. Helm B: **Gateway to health: Taijiquan and traditional Chinese medicine.** *Taijiqua J* 2002, **8:**12.
15. Lan C, Chen SY, Wong MK, Lai JS: **Tai Chi training for patients with coronary heart disease.** *Med Sport Sci* 2008, **52:**182–194.
16. Yeh GY, Mietus JE, Peng CK, Phillips RS, Davis RB, Wayne PM, Goldberger AL, Thomas RJ: **Enhancement of sleep stability with Tai Chi exercise in**

chronic heart failure: preliminary findings using an ECG-based spectrogram method. *Sleep Med* 2008, 9:527–536.
17. Fontana JA, Colella C, Baas LS, Ghazi F: T'ai Chi Chih as an intervention for heart failure. *Nurs Clin North Am* 2000, 35:1031–1046.
18. Barrow DE, Bedford A, Ives G, O'Toole L, Channer KS: An evaluation of the effects of Tai Chi Chuan and Chi Kung training in patients with symptomatic heart failure: a randomised controlled pilot study. *Postgrad Med J* 2007, 83:717–721.
19. Young DR, Appel LJ, Jee S, Miller ER 3rd: The effects of aerobic exercise and T'ai Chi on blood pressure in older people: results of a randomized trial. *J Am Geriatr Soc* 1999, 47:277–284.
20. Yeh GY, Wang C, Wayne PM, Phillips RS: The effect of tai chi exercise on blood pressure: a systematic review. *Prev Cardiol* 2008, 11:82–89.
21. Wolf SL, Sattin RW, Kutner M, O'Grady M, Greenspan AI, Gregor RJ: Intense tai chi exercise training and fall occurrences in older, transitionally frail adults: a randomized, controlled trial. *J Am Geriatr Soc* 2003, 51:1693–1701.
22. Lan C, Lai JS, Chen SY, Wong MK: Tai Chi Chuan to improve muscular strength and endurance in elderly individuals: a pilot study. *Arch Phys Med Rehabil* 2000, 81:604–607.
23. Wu G, Zhao F, Zhou X, Wei L: Improvement of isokinetic knee extensor strength and reduction of postural sway in the elderly from long-term Tai Chi exercise. *Arch Phys Med Rehabil* 2002, 83:1364–1369.
24. Wu G: Evaluation of the effectiveness of Tai Chi for improving balance and preventing falls in the older population–a review. *J Am Geriatr Soc* 2002, 50:746–754.
25. Wayne PM, Krebs DE, Wolf SL, Gill-Body KM, Scarborough DM, McGibbon CA, Kaptchuk TJ, Parker SW: Can Tai Chi improve vestibulopathic postural control? *Arch Phys Med Rehabil* 2004, 85:142–152.
26. Wayne PM, Kiel DP, Krebs DE, Davis RB, Savetsky-German J, Connelly M, Buring JE: The effects of Tai Chi on bone mineral density in postmenopausal women: a systematic review. *Arch Phys Med Rehabil* 2007, 88:673–680.
27. Wang C, Roubenoff R, Lau J, Kalish R, Schmid CH, Tighiouart H, Rones R, Hibberd PL: Effect of Tai Chi in adults with rheumatoid arthritis. *Rheumatology (Oxford)* 2005, 44:685–687.
28. Hartman CA, Manos TM, Winter C, Hartman DM, Li B, Smith JC: Effects of T'ai Chi training on function and quality of life indicators in older adults with osteoarthritis. *J Am Geriatr Soc* 2000, 48:1553–1559.
29. Wang C, Schmid CH, Rones R, Kalish R, Yinh J, Goldenberg DL, Lee Y, McAlindon T: A randomized trial of tai chi for fibromyalgia. *N Engl J Med* 2010, 363:743–754.
30. Li JX, Hong Y, Chan KM: Tai chi: physiological characteristics and beneficial effects on health. *Br J Sports Med* 2001, 35:148–156.
31. Wayne PM, Walsh JN, Taylor-Piliae RE, Wells RE, Papp KV, Donovan NJ, Yeh GY: Effect of Tai Chi on Cognitive Performance in Older Adults: Systematic Review and Meta-Analysis. *J Am Geriatr Soc* 2014, 62:25–39.
32. Wang C, Collet JP, Lau J: The effect of Tai Chi on health outcomes in patients with chronic conditions: a systematic review. *Arch Intern Med* 2004, 164:493–501.
33. Wayne PM, Fuerst ML: *The Harvard Medical School Guide to Tai Chi.* United States of America: Shambhala Publications, Inc.; 2013.
34. Yan JH, Guo YZ, Yao HM, Pan L: Effects of Tai Chi in patients with chronic obstructive pulmonary disease: preliminary evidence. *PLoS One* 2013, 8:e61806.
35. Ding M, Zhang W, Li K, Chen X: Effectiveness of T'ai Chi and qigong on chronic obstructive pulmonary disease: a systematic review and meta-analysis. *J Altern Complement Med* 2014, 20:79–86.
36. Wayne P, Kaptchuk T: Challenges inherent to Tai Chi research: Part I–Tai Chi as a complex multi-component intervention. *J Altern Complement Med* 2008, 14:95–102.
37. Lacasse Y, Goldstein R, Lasserson TJ, Martin S: Pulmonary rehabilitation for chronic obstructive pulmonary disease. *Cochrane Database Syst Rev* 2006, 4, CD003793.
38. Lacasse Y, Martin S, Lasserson TJ, Goldstein RS: Meta-analysis of respiratory rehabilitation in chronic obstructive pulmonary disease. A Cochrane systematic review. *Eura Medicophys* 2007, 43:475–485.
39. Ries AL, Bauldoff GS, Carlin BW, Casaburi R, Emery CF, Mahler DA, Make B, Rochester CL, Zuwallack R, Herrerias C: Pulmonary Rehabilitation: Joint ACCP/AACVPR Evidence-Based Clinical Practice Guidelines. *Chest* 2007, 131(Suppl 5):4S–42S.
40. Watz H, Waschki B, Meyer T, Magnussen H: Physical activity in patients with COPD. *Eur Respir J* 2009, 33:262–272.
41. Waschki B, Kirsten A, Holz O, Muller KC, Meyer T, Watz H, Magnussen H: Physical activity is the strongest predictor of all-cause mortality in patients with COPD: a prospective cohort study. *Chest* 2011, 140:331–342.
42. Garcia-Aymerich J, Lange P, Benet M, Schnohr P, Anto JM: Regular physical activity reduces hospital admission and mortality in chronic obstructive pulmonary disease: a population based cohort study. *Thorax* 2006, 61:772–778.
43. Esteban C, Arostegui I, Aburto M, Moraza J, Quintana JM, Aizpiri S, Basualdo LV, Capelastegui A: Influence of changes in physical activity on frequency of hospitalization in chronic obstructive pulmonary disease. *Respirology (Carlton, Vic)* 2014, 19:330–338.
44. Spruit MA, Singh SJ, Garvey C, ZuWallack R, Nici L, Rochester C, Hill K, Holland AE, Lareau SC, Man WD, Pitta F, Sewell L, Raskin J, Bourbeau J, Crouch R, Franssen FM, Casaburi R, Vercoulen JH, Vogiatzis I, Gosselink R, Clini EM, Effing TW, Maltais F, van der Palen J, Troosters T, Janssen DJ, Collins E, Garcia-Aymerich J, Brooks D, Fahy BF, *et al*: An official American Thoracic Society/European Respiratory Society statement: key concepts and advances in pulmonary rehabilitation. *Am J Respir Crit Care Med* 2013, 188:e13–e64.
45. ZuWallack R, Hedges H: Primary care of the patient with chronic obstructive pulmonary disease-part 3: pulmonary rehabilitation and comprehensive care for the patient with chronic obstructive pulmonary disease. *Am J Med* 2008, 121(Suppl 7):S25–S32.
46. Pitta F, Troosters T, Spruit MA, Probst VS, Decramer M, Gosselink R: Characteristics of physical activities in daily life in chronic obstructive pulmonary disease. *Am J Respir Crit Care Med* 2005, 171:972–977.
47. Van Remoortel H, Hornikx M, Demeyer H, Langer D, Burtin C, Decramer M, Gosselink R, Janssens W, Troosters T: Daily physical activity in subjects with newly diagnosed COPD. *Thorax* 2013, 68:962–963.
48. Butts JF, Belfer MH, Gebke KB: Exercise for patients with COPD: an integral yet underutilized intervention. *Phys Sportsmed* 2013, 41:49–57.
49. Fontana JA, Colella C, Wilson BR, Baas L: The energy costs of a modified form of T'ai Chi exercise. *Nurs Res* 2000, 49:91–96.
50. Lan C, Chen SY, Lai JS: The exercise intensity of Tai Chi Chuan. *Med Sport Sci* 2008, 52:12–19.
51. Wang C, Schmid C, Hibberd P, Kalish R, Roubenoff R, Rones R, McAlinden T: Tai Chi is effective in treating knee osteoarthritis: a randomized controlled trial. *Arthritis Rheum* 2009, 61:1545–1553.
52. Yeh GY, Roberts DH, Wayne PM, Davis RB, Quilty MT, Phillips RS: Tai Chi Exercise for Patients With Chronic Obstructive Pulmonary Disease: A Pilot Study. *Respir Care* 2010, 55:1475–1482.
53. Wolf SL, O'Grady M, Easley KA, Guo Y, Kressig RW, Kutner M: The influence of intense Tai Chi training on physical performance and hemodynamic outcomes in transitionally frail, older adults. *J Gerontol A Biol Sci Med Sci* 2006, 61:184–189.
54. Lotters F, van Tol B, Kwakkel G, Gosselink R: Effects of controlled inspiratory muscle training in patients with COPD: a meta-analysis. *Eur Respir J* 2002, 20:570–576.
55. Weiner P, Magadle R, Beckerman M, Weiner M, Berar-Yanay N: Comparison of specific expiratory, inspiratory, and combined muscle training programs in COPD. *Chest* 2003, 124:1357–1364.
56. O'Brien K, Geddes EL, Reid WD, Brooks D, Crowe J: Inspiratory muscle training compared with other rehabilitation interventions in chronic obstructive pulmonary disease: a systematic review update. *J Cardiopulm Rehabil Prev* 2008, 28:128–141.
57. Gosselink R: Breathing techniques in patients with chronic obstructive pulmonary disease (COPD). *Chron Respir Dis* 2004, 1:163–172.
58. Cahalin LP, Braga M, Matsuo Y, Hernandez ED: Efficacy of diaphragmatic breathing in persons with chronic obstructive pulmonary disease: a review of the literature. *J Cardiopulm Rehabil* 2002, 22:7–21.
59. Dechman G, Wilson CR: Evidence underlying breathing retraining in people with stable chronic obstructive pulmonary disease. *Phys Ther* 2004, 84:1189–1197.
60. van Gestel AJ, Steier J: Autonomic dysfunction in patients with chronic obstructive pulmonary disease (COPD). *J Thorac Dis* 2010, 2:215–222.
61. Cheng ST, Wu YK, Yang MC, Huang CY, Huang HC, Chu WH, Lan CC: Pulmonary rehabilitation improves heart rate variability at peak exercise, exercise capacity and health-related quality of life in chronic obstructive pulmonary disease. *Heart Lung* 2014, 43(3):249–255.
62. Pbert L, Madison JM, Druker S, Olendzki N, Magner R, Reed G, Allison J, Carmody J: Effect of mindfulness training on asthma quality of life and lung function: a randomised controlled trial. *Thorax* 2012, 67:769–776.

63. Daubenmier J, Sze J, Kerr CE, Kemeny ME, Mehling W: **Follow your breath: respiratory interoceptive accuracy in experienced meditators.** *Psychophysiology* 2013, **50:**777–789.
64. Young EC, Brammer C, Owen E, Brown N, Lowe J, Johnson C, Calam R, Jones S, Woodcock A, Smith JA: **The effect of mindfulness meditation on cough reflex sensitivity.** *Thorax* 2009, **64:**993–998.
65. Greeson JM: **Mindfulness Research Update: 2008.** *Compl Health Pract Rev* 2009, **14:**10–18.
66. Lacasse Y, Rousseau L, Maltais F: **Prevalence of depressive symptoms and depression in patients with severe oxygen-dependent chronic obstructive pulmonary disease.** *J Cardiopulm Rehabil* 2001, **21:**80–86.
67. Biorkovee TNS: **Credibility of analogue therapy rationales.** *J Behav Ther Exp Psychiatry* 1972, **3:**257–260.
68. Yeh GY, McCarthy EP, Wayne PM, Stevenson LW, Wood MJ, Forman D, Davis RB, Phillips RS: **Tai chi exercise in patients with chronic heart failure: a randomized clinical trial.** *Arch Intern Med* 2011, **171:**750–757.
69. American Thoracic Society and European Respiratory Society: **Standards for Diagnosis and Management of Patients with COPD.** http://www.thoracic.org/clinical/copd-guidelines/resources/copdpatient.pdf.
70. Global Initiative for Chronic Obstructive Lung Disease: **What You Can Do About a Lung Disease Called COPD.** http://www.goldcopd.org/uploads/users/files/GOLD_Patient_RevJan10.pdf.
71. **Living Well with COPD A plan of action for your life.** http://www.livingwellwithcopd.com/en/about.html.
72. American Thoracic Society: **Patient Information Series & Patient Health Series.** http://patients.thoracic.org.
73. **COPD Foundation.** http://www.copdfoundation.org/Home/Understand¬ing-COPD/What-is-COPD.aspx.
74. CHEST American College of CHEST Physician: **Patient Education Resources.** http://www.chestnet.org/Publications/Other-Publications/Patient-Education-Guides.
75. Chia M: *Tan Tien Chi Kung. Revised edition.* Rochester: Destiny Books; 2004.
76. Yang Y: *Taijiquan: the Art of Nuturing, the Science of Power.* Champaign: Zhen Wu Publications; 2005.
77. Frantzis B: *Opening the Energy Gates of Your Body.* 2nd edition. Berkeley: Blue Snake Books; 2005.
78. Yang J-M: *The Essence of Taiji Qigong: The Internal Foundation of Taijiquan.* 2nd edition. Boston: YMAA Publication Center; 1998.
79. Guyatt GH, Sullivan MJ, Thompson PJ, Fallen EL, Pugsley SO, Taylor DW, Berman LB: **The 6-minute walk: a new measure of exercise capacity in patients with chronic heart failure.** *Can Med Assoc J* 1985, **132:**919–923.
80. Gerardi DA, Lovett L, Benoit-Connors ML, Reardon JZ, ZuWallack RL: **Variables related to increased mortality following out-patient pulmonary rehabilitation.** *Eur Respir J* 1996, **9:**431–435.
81. Polkey MI, Spruit MA, Edwards LD, Watkins ML, Pinto-Plata V, Vestbo J, Calverley PM, Tal-Singer R, Agusti A, Bakke PS, Coxson HO, Lomas DA, MacNee W, Rennard S, Silverman EK, Miller BE, Crim C, Yates J, Wouters EF, Celli B, Evaluation of COPD Longitudinally to Identify Predictive Surrogate Endpoints (ECLIPSE) Study Investigators: **Six-minute-walk test in chronic obstructive pulmonary disease: minimal clinically important difference for death or hospitalization.** *Am J Respir Crit Care Med* 2013, **187:**382–386.
82. Schunemann HJ, Puhan M, Goldstein R, Jaeschke R, Guyatt GH: **Measurement properties and interpretability of the Chronic respiratory disease questionnaire (CRQ).** *COPD* 2005, **2:**81–89.
83. Cote CG, Pinto-Plata V, Kasprzyk K, Dordelly LJ, Celli BR: **The 6-min walk distance, peak oxygen uptake, and mortality in COPD.** *Chest* 2007, **132:**1778–1785.
84. Jones CJ, Rikli RE, Beam WC: **A 30-s chair-stand test as a measure of lower body strength in community-residing older adults.** *Res Q Exerc Sport* 1999, **70:**113–119.
85. Jones CJ, Rikli RE, Max J, Noffal G: **The reliability and validity of a chair sit-and-reach test as a measure of hamstring flexibility in older adults.** *Res Q Exerc Sport* 1998, **69:**338–343.
86. Verrill D, Barton C, Beasley W, Lippard WM: **The effects of short-term and long-term pulmonary rehabilitation on functional capacity, perceived dyspnea, and quality of life.** *Chest* 2005, **128:**673–683.
87. Hajiro T, Nishimura K, Tsukino M, Ikeda A, Koyama H, Izumi T: **Comparison of discriminative properties among disease-specific questionnaires for measuring health-related quality of life in patients with chronic obstructive pulmonary disease.** *Am J Respir Crit Care Med* 1998, **157:**785–790.
88. Celli BR, Cote CG, Marin JM, Casanova C, Montes de Oca M, Mendez RA, Pinto Plata V, Cabral HJ: **The body-mass index, airflow obstruction, dyspnea, and exercise capacity index in chronic obstructive pulmonary disease.** *N Engl J Med* 2004, **350:**1005–1012.
89. Wigal JK, Creer TL, Kotses H: **The COPD Self-Efficacy Scale.** *Chest* 1991, **99:**1193–1196.
90. Radloff L: **The CES-D scale: a self-report depression scale for research in the general population.** *Appl Psychol Meas* 1977, **1:**385–401.
91. van Manen JG, Bindels PJ, Dekker FW, Ijzermans CJ, van der Zee JS, Schade E: **Risk of depression in patients with chronic obstructive pulmonary disease and its determinants.** *Thorax* 2002, **57:**412–416.
92. Himmelfarb S, Murrell SA: **Reliability and validity of five mental health scales in older persons.** *J Gerontol* 1983, **38:**333–339.
93. Rose M, Bjorner JB, Becker J, Fries JF, Ware JE: **Evaluation of a preliminary physical function item bank supported the expected advantages of the Patient-Reported Outcomes Measurement Information System (PROMIS).** *J Clin Epidemiol* 2008, **61:**17–33.
94. Garcia SF, Cella D, Clauser SB, Flynn KE, Lad T, Lai JS, Reeve BB, Smith AW, Stone AA, Weinfurt K: **Standardizing patient-reported outcomes assessment in cancer clinical trials: a patient-reported outcomes measurement information system initiative.** *J Clin Oncol* 2007, **25:**5106–5112.
95. Cohen S, Kamarck T, Mermelstein R: **A global measure of perceived stress.** *J Health Soc Behav* 1983, **24:**385–396.
96. Zimet GDPS, Farley GK, Werkman S, Berkoff KA: **Psychometric characteristics of the multidimensional scale of preceived social support.** *J Pers Assess* 1990, **55:**610–617.
97. Miller MRHJ, Brusasco V, Burgos F, Casaburi R, Coates A, Crap R, Enright P, der Grinten CPM C, Gustafsson P, Jensen R, Johnson DC, Macintyre N, McKay R, Navajas D, Pedersen OF, Pellegrino R, Viegi G, Wanger J: **"ATS/ERS Task Force: Standardisation of Lung Function Testing" Standardisation of spirometry.** *Eur Respir J* 2005, **26:**319–338.
98. Albuquerque AL, Nery LE, Villaca DS, Machado TY, Oliveira CC, Paes AT, Neder JA: **Inspiratory fraction and exercise impairment in COPD patients GOLD stages II-III.** *Eur Respir J* 2006, **28:**939–944.
99. Stewart AL, Mills KM, King AC, Haskell WL, Gillis D, Ritter PL: **CHAMPS physical activity questionnaire for older adults: outcomes for interventions.** *Med Sci Sports Exerc* 2001, **33:**1126–1141.
100. Moy ML, Matthess K, Stolzmann K, Reilly J, Garshick E: **Free-living physical activity in COPD: assessment with accelerometer and activity checklist.** *J Rehabil Res Dev* 2009, **46:**277–286.
101. de Torres JP, Pinto-Plata V, Ingenito E, Bagley P, Gray A, Berger R, Celli B: **Power of outcome measurements to detect clinically significant changes in pulmonary rehabilitation of patients with COPD.** *Chest* 2002, **121:**1092–1098.
102. Puhan MA, Mador MJ, Held U, Goldstein R, Guyatt GH, Schunemann HJ: **Interpretation of treatment changes in 6-minute walk distance in patients with COPD.** *Eur Respir J* 2008, **32:**637–643.
103. Holland AE, Hill CJ, Rasekaba T, Lee A, Naughton MT, McDonald CF: **Updating the minimal important difference for six-minute walk distance in patients with chronic obstructive pulmonary disease.** *Arch Phys Med Rehabil* 2010, **91:**221–225.
104. Redelmeier DA, Guyatt GH, Goldstein RS: **Assessing the minimal important difference in symptoms: a comparison of two techniques.** *J Clin Epidemiol* 1996, **49:**1215–1219.
105. Conboy LA, Noggle JJ, Frey JL, Kudesia RS, Khalsa SB: **Qualitative evaluation of a high school yoga program: feasibility and perceived benefits.** *Explore (New York, NY)* 2013, **9:**171–180.
106. Kerr CE, Shaw JR, Conboy LA, Kelley JM, Jacobson E, Kaptchuk TJ: **Placebo acupuncture as a form of ritual touch healing: a neurophenomenological model.** *Conscious Cogn* 2011, **20:**784–791.
107. Kaptchuk TJ, Shaw J, Kerr CE, Conboy LA, Kelley JM, Csordas TJ, Lembo AJ, Jacobson EE: **"Maybe I made up the whole thing": placebos and patients' experiences in a randomized controlled trial.** *Cult Med Psychiatry* 2009, **33:**382–411.
108. Conboy L, Quilty MT, Kerr C, Shaw J, Wayne P: **A qualitative analysis of adolescents' experiences of active and sham Japanese-style acupuncture protocols administered in a clinical trial.** *J Altern Complement Med* 2008, **14:**699–705.

doi:10.1186/1745-6215-15-337
Cite this article as: Yeh *et al.*: Tai chi mind-body exercise in patients with COPD: study protocol for a randomized controlled trial. *Trials* 2014 **15**:337.
