# Supplementary material for: The Effects of Traditional Chinese Exercise in Patients with Chronic Obstructive Pulmonary Disease: A Meta-Analysis
Source: PLoS One. 2016 Sep 2;11(9):e0161564. doi: 10.1371/journal.pone.0161564 (PMC5010221; doi:10.1371/journal.pone.0161564)
Supplement: S2 Appendix — (ZIP) [file pone.0161564.s002.zip › S2 Appendix Reasons for excluded articles/Article2 .pdf]

Sun-style T'ai Chi improves walking endurance and health-related quality of life in people with COPD

Synopsis

Summary of: Leung RWM, et al (2013) Short-form Sun-style t'ai chi as an exercise training modality in people with COPD. *Eur Respir J* 41: 1051–1057. [Prepared by Kylie Hill, CAP Editor.]

Question: Does short-form Sun-style T'ai Chi (SSTC) change exercise capacity, balance, physical performance, quadriceps strength, health-related quality of life (HRQoL) or mood in patients with chronic obstructive pulmonary disease (COPD)? ***Design***: Randomised controlled trial with concealed allocation and blinding of outcome assessors. ***Setting***: The out-patient department of a hospital in Sydney, Australia. ***Participants***: Adults with stable COPD were included if they had not participated in exercise training in the previous 12 months, had no significant co-morbidities that precluded participation in SSTC and did not require supplemental oxygen during exercise. Randomisation allocated 22 to the intervention group and 20 to the control group. ***Interventions***: Participants in the intervention group attended two supervised 1-hour sessions of SSTC training each week, for 12 weeks. Training intensity was titrated to achieve a moderate dyspnoea by encouraging participants to imagine pushing against a resistance, perform deeper squats or with the use of wrist weights. Participants were also encouraged to complete 30 minutes of SSTC each day that they did not attend a supervised session and were provided with a training booklet and DVD to facilitate home training. Those in the control group continued with usual medical care. ***Outcome measures***: The primary outcome measure was time walked during the endurance shuttle walk test (ESWT) at 12 weeks. ***Results***: A total of 38 participants completed the study. On completion of the training period, greater gains were seen in the intervention group compared with the control group in time walked during the ESWT (348 sec; 95% confidence interval [CI], 186 to 510 sec). Significant between group differences, in favour of the intervention group, were also seen in measures of balance, physical performance, quadriceps force, HRQoL and anxiety. ***Conclusion:*** The use of SSTC is an effective intervention to improve several outcomes in people with COPD, including walking endurance, HRQoL and quadriceps force.

Commentary

Chronic obstructive pulmonary disease (COPD) is a condition characterised by impaired pulmonary function and reduced exercise capacity and health-related quality of life (HRQoL). There is compelling evidence that pulmonary rehabilitation is effective at improving exercise capacity, physical function and HRQoL in people with stable COPD (Lacasse et al 2006) and following exacerbations (Puhan et al 2011). Appropriate exercise reconditioning is essential for successful pulmonary rehabilitation but exercise outcomes vary depending on the mechanism of exercise limitation (Plankeel et al 2005). The mode of exercise training should be versatile and tailored to an individual's needs. The study by Leung et al (2013) describes a Sun-Style T'ai Chi (SSTC) exercise program which resulted in a clinically important increase in endurance, balance and HRQoL scores. The Incremental Shuttle Walk Test (ISWT) was used to establish SSTC exercise intensity, which was assessed as moderate. The commendable aspect of the study was adding resistance loading to the wrists during T'ai Chi practice to ensure standardisation of exercise intensity. Most T'ai Chi studies allow their subjects to practice traditional, relaxed, smooth and rhythmical movements. The SSTC combined a 'hard and soft' form of Chinese martial arts, which ensured a training intensity high enough to yield physiological training benefit, which satisfies the essential principle of western exercise training. The focus on rhythmic breathing during SSTC may have expedited better diaphragmatic control during exercise in people with COPD.

A key goal of pulmonary rehabilitation is to encourage the patient to maintain an active lifestyle. If the practice of T'ai Chi arouses sufficient interest in people with COPD for them to adopt this as a daily exercise of moderate intensity, future work should focus on how SSTC could be incorporated in pulmonary rehabilitation.

Alice Jones
Department of Physiotherapy, The University of Sydney, Australia
School of Rehabilitation Sciences, Griffith University, Australia

References

Lacasse Y et al (2006) *Cochrane Database Syst Rev* 4: CD003793.

Plankeel J et al (2005) *Chest* 127:110–116.

Puhan M et al (2011) *Cochrane Database Syst Rev* 10: CD003793.

Troosters T et al (2005) *Am J Respir Crit Care Med* 172: 19–38.
