# Supplementary material for: The Effects of Traditional Chinese Exercise in Patients with Chronic Obstructive Pulmonary Disease: A Meta-Analysis
Source: PLoS One. 2016 Sep 2;11(9):e0161564. doi: 10.1371/journal.pone.0161564 (PMC5010221; doi:10.1371/journal.pone.0161564)
Supplement: S2 Appendix — (ZIP) [file pone.0161564.s002.zip › S2 Appendix Reasons for excluded articles/Article3 .pdf]

Li et al. BMC Complementary and Alternative Medicine 2012, **12**:197
http://www.biomedcentral.com/1472-6882/12/197

BMC Complementary & Alternative Medicine

RESEARCH ARTICLE Open Access

Effects of comprehensive therapy based on traditional Chinese medicine patterns in stable chronic obstructive pulmonary disease: a four-center, open-label, randomized, controlled study

Su-yun Li[1], Jian-sheng Li[1,2*], Ming-hang Wang[1], Yang Xie[2], Xue-qing Yu[1], Zi-kai Sun[3], Li-jun Ma[4], Wei Zhang[5], Hai-long Zhang[2], Fan Cao[2] and Ying-chao Pan[2]

Abstract

Background: Traditional Chinese medicine (TCM) has been used to treat chronic obstructive pulmonary disease (COPD) for many years. This study aimed to evaluate the efficacy and safety of the comprehensive therapy based on the three common TCM patterns in stable COPD patients.

Methods: A four-center, open-label randomized controlled method was conducted. A total of 352 patients were divided into the trial group (n = 176, treated with conventional Western medicine and Bu-Fei Jian-Pi granules, Bu-Fei Yi-Shen granules, and Yi-Qi Zi-Shen granules based on the TCM patterns respectively) and the control group (n = 176, treated with conventional Western medicine). The frequency and duration of acute exacerbation, lung function, clinical symptoms, 6-minute walking distance (6MWD), dyspnea scale and quality of life were observed during a 6-month treatment period and at a further 12-month follow-up.

Results: A total of 306 patients completed the study fully. The full analysis set (FAS) population was 350 and the per-protocol analysis set (PPS) population was 306. After the 6-month treatment and 12-month follow-up, there were significant differences between the trial and control group in the following: frequency of acute exacerbation (FAS: P = 0.000; PPS: P = 0.000); duration of acute exacerbation (FAS: P = 0.000; PPS: P = 0.001); FEV1 (FAS: P = 0.007; PPS: P = 0.008); symptoms (FAS: P = 0.001; PPS: P = 0.001); 6MWD (FAS: P = 0.045; PPS: P = 0.042); dyspnea scale (FAS: P = 0.002; PPS: P = 0.004); and physical domain (FAS: P = 0.000; PPS: P = 0.000), psychological domain (FAS: P = 0.008; PPS: P = 0.011), social domain (FAS: P = 0.001; PPS: P = 0.000) and environment domain (FAS: P = 0.015; PPS: P = 0.009) of the WHOQOL-BREF questionnaire. There were no differences between the trial and control group in FVC, FEV1% and adverse events.

Conclusions: Based on the TCM patterns, Bu-Fei Jian-Pi granules, Bu-Fei Yi-Shen granules and Yi-Qi Zi-Shen granules have beneficial effects on measured outcomes in stable COPD patients over the 6-month treatment and 12-month follow-up, with no relevant between-group differences in adverse events.

Trial Registration: This trial was registered at Chinese Clinical Trial Register Center, ChiCTR-TRC-11001406.

Keywords: Chronic obstructive pulmonary disease, Traditional Chinese medicine pattern, Clinical trial, Bu-Fei Jian-Pi granules, Bu-Fei Yi-Shen granules, Yi-Qi Zi-Shen granules

* Correspondence: li_js8@163.com
[1]Department of Respiratory Diseases, The First Affiliated Hospital of Henan University of Traditional Chinese Medicine, No. 19 Renmin Road, Zhengzhou, PR China
[2]The Geriatric Department, Henan University of Traditional Chinese Medicine, No. 1 Jinshui Road, Zhengzhou, PR China
Full list of author information is available at the end of the article

BioMed Central

© 2012 Li et al.; licensee BioMed Central Ltd. This is an Open Access article distributed under the terms of the Creative Commons Attribution License (http://creativecommons.org/licenses/by/2.0), which permits unrestricted use, distribution, and reproduction in any medium, provided the original work is properly cited.

Background

Chronic obstructive pulmonary disease (COPD) is a major public health problem throughout the world. The high prevalence, morbidity, mortality and economic burdens of COPD are increasing steadily. The projection for 2020 indicates that COPD will become the third leading cause of death worldwide [1]. Because of tobacco smoking, solid-fuel use, and other reasons, an estimated 65 million people will die of COPD between 2003 and 2033 in China [2]. Therefore, designing strategies and treatments for COPD is important. In light of evidence-based medicine, short-acting bronchodilators, long-acting bronchodilators, inhaled glucocorticosteroids, and low-dose, slow-release theophylline are the established interventions for treating COPD patients [3]. However, it is difficult to improve people's symptoms without suffering too many side effects or adverse events [4].

The remarkable longevity and current popularity of traditional Chinese medicine (TCM) for COPD implies its potential advantages in ameliorating symptoms, reducing the frequency of acute exacerbation, and improving quality of life on the basis of different patterns and stages [5,6]. There is limited evidence for randomized controlled trials on comprehensive TCM interventions, especially based on the TCM patterns. Therefore it is difficult to fully reflect the efficacy, characteristics and advantages of TCM [7]. The TCM pattern is a specific stratification of a disease according to a group of symptoms, which can be regarded as a summary of the body's condition at a certain stage in a disease process [8]. A potential way of studying COPD is by adopting the method of TCM patterns in clinical practice. According to our previous study, there are three common TCM patterns of stable COPD, and there is one specific herbal intervention responding to each pattern, which reflects the concepts of individualized therapy of TCM [9]. Therefore, we performed a multi-center randomized controlled study that had been carried out to evaluate the efficacy and safety of comprehensive interventions based on the three TCM patterns in patients with stable COPD.

Methods

Participants

Diagnostic criteria

COPD was diagnosed by the Global Strategy for the Diagnosis, Management, and Prevention of COPD, and the Chinese Treatment Guidelines of COPD [10,11].

Traditional Chinese medicine pattern criteria

The TCM pattern criteria of stable COPD were established by literature analysis, clinical investigation, expert counseling, scientific assessment and clinical application [9,12]. There are three common patterns as follows.

Pattern of lung-spleen qi deficiency: Symptoms of lung-spleen qi deficiency included the following: (1) coughing and shortness of breath which are worse when active; (2) lassitude and spontaneous sweating, which are worse when active; (3) being prone to catching a cold; (4) poor appetite or eating less; (5) bloating in the gastric cavity, abdominal distension or loose stools; and (6) an enlarged tongue with a white or greasy fur and deep thready pulse, deep slow pulse or thready weak pulse. As long as the patients showed any two of symptoms (1), (2), or (3), along with any two of symptoms (4), (5), or (6), the pattern of lung-spleen qi deficiency was diagnosed.

Pattern of lung-kidney qi deficiency: Symptoms of lung-kidney qi deficiency included the following: (1) panting, shortness of breath, which are worse when active; (2) lassitude and spontaneous sweating, which are worse when active; (3) being prone to catching a cold; (4) weakness in the lower back and knees; (5) tinnitus, vertigo, or asthenia facial edema; (6) profuse urine, frequent urination at night, or urine released with coughing; and (7) a pale tongue with white fur, deep thready pulse or thready weak pulse. As long as the patients showed any two of symptoms (1), (2), or (3), along with any two of symptoms (4), (5), (6), or (7), the pattern of lung-kidney qi deficiency was diagnosed.

Pattern of lung-kidney qi and yin deficiency: Symptoms of lung-kidney qi and yin deficiency included the following: (1) panting and shortness of breath, which are worse when active; (2) lassitude, which is worse when active; (3) being prone to catching a cold; (4) weakness in the lower back and knees; (5) tinnitus and dizziness; (6) a dry cough or scanty sputum and difficult in spitting; (7)spontaneous sweating or night sweating; (8) feverishness in the palms and soles; and (9) a pale or red tongue with thin and little fur, and a thready pulse, thready weak pulse or thready rapid pulse. As long as the patients showed any two of symptoms (1), (2), or (3), along with any one of symptoms (4), or (5), and any two of the symptoms (6), (7), (8), or (9), the pattern of lung-kidney qi and yin deficiency was diagnosed.

Inclusion criteria

Patients were included in the study according to the following: (1) patients met the diagnostic criteria; (2) patients met the TCM pattern criteria of stable COPD (TCM diagnosis); (3) patients were stable and met the diagnosis of mild to severe COPD (Global Initiative for Chronic Obstructive Lung Disease, GOLD 1,2,3); (4) patients were aged between 40 to 80 years; (5) patients underwent a two-week wash-out period prior to randomization; (6) patients had no experience in other interventional trials in the previous 1 month; and (7) patients should receive the treatment voluntarily and sign informed consent.

Exclusion criteria

Patients were excluded from the study for the following reasons: (1) acute exacerbation of COPD or very severe COPD (GOLD 4); (2) female patients were in pregnant or breast-feeding; (3) confusion, dementia or any type of mental illness that rendered patients unable to understand the nature, scope and possible consequences of the study; (4) patients were complicated by severe heart failure (Grade II or IV New York Heart Association [NYHA] Functional Classification); (5) patients were complicated with bronchial asthma, bronchiectasis, active tuberculosis, pulmonary embolism or diffuse panbronchiolitis; (6) patients were complicated with a neuromuscular disorder, which affected the respiration; (7) patients with serious hepatic and renal diseases, such as liver cirrhosis, portal hypertension, bleeding of varicose veins, dialysis, or renal transplantation; (8) congenital or acquired immune deficiency; (9) participation in other clinical intervention research; and (10) allergies to treatment drugs.

Ethics and trial registration

The study was approved by the Ethical Research Committees of The First Affiliated Hospital of Henan University of Traditional Chinese Medicine (batch number: YFYKTLL2007-1). The study was registered at the Chinese Clinical Trial Register Center (ChiCTR-TRC-11001406).

Entry procedure

Patients with stable COPD were enrolled from either the out-patient department or open recruitment and were observed in four centers, including the First Affiliated Hospital of Henan University of Traditional Chinese Medicine, Jiangsu Provincial Hospital of Traditional Chinese Medicine, Henan Provincial People's Hospital and the Affiliated Hospital of Shandong University of Traditional Chinese Medicine. All patients signed the informed consent before inclusion.

Study design

Sample Size

A total of 352 cases were divided into the trial group (n=176) and the control group (n=176). The frequency of acute exacerbation was considered as the primary outcome. From a previous study, the number of the exacerbation frequency decreased by 0.44 times every half year by the TCM comprehensive interventions compared with the Western medicine treatment [13]. We assumed that when the number of exacerbation frequency was decreased at least by 1 times between the two groups and the standard deviation was 1.5 times/year, the efficacy of TCM comprehensive interventions could be reflected and had promotional value. The formulae ($\frac{2(\mu_\alpha+\mu_\beta)^2\sigma^2}{\delta^2}$) was based on a comparison between the equal numbers of a two sample mean. The two-sided alpha level was 0.05, and the beta level was 0.10. The δ value was 1, the σ value was 1.5, the μ_α value was 1.96, the μ_β value was 1.28. Using the calculation, $N=2^*(1.96+1.28)^{2}*1.5^2/1.0^2$, the sample size in each group was approximately 48. If the severity of airflow limitation in COPD (GOLD 1, 2, 3) was considered as the stratification factor, and the stratification factor was 3, then 144 (48*3) patients were required in each group, and the sample size in the two groups would be 288 (144*2). Allowing for a 15% dropout rate over the course of the study, the sample size would be approximately 332 (288+288*0.15). The sample size was inflated to allow for the balance of the research centers, with two patients were added to each group, and therefore the sample size was 336 (332+2*2). To avoid the negative effect of accidents in clinical implementation, four patients were also added in each research center. The number of research centers was four, and therefore, the final sample size was 352 (336+4*4), with 176 patients in each group.

Randomization

A stratified and block randomization design was adopted. The number of the groups was 2 and the distribution ratio was 1-to-1. Considering the long time for treatment observation, the process was divided into more than one block, and the length of the block was four. The number of center hierarchical levels was four. A random number from 001 to 352 was generated by SAS 9.2 and saved in a sealed envelope by an independent clinical statistician. Treatment allocation occurred when the participant met the inclusion criteria and signed the informed consent form. In the event of a clinical emergency, the individual's randomization code and group allocation could be identified by the emergency envelope as soon as possible. The randomization design was provided by the DME department of Guangzhou University of Traditional Chinese Medicine.

The study was an open-label trial, however, some measures were taken to strengthen quality control. An investigator separate from all of the clinical researchers was assigned in each research center as the contact person who preserved and recorded the randomization information. Therefore, the clinical researchers did not have any effect on enrollment or randomization. Meanwhile outcome assessments were made by an independent clinical statistician blinded to group allocation and uninvolved in providing intervention or management.

Interventions

Patients in the control group were given conventional Western medicine treatment based on the classes of

medications recommended by the Global Initiative for Chronic Obstructive Lung Disease (GOLD) and Chinese Treatment Guidelines of COPD [10,11]. The specific therapies were shown in Table 1. For the trial group, which was given conventional Western medicine treatment, patients were additionally given Bu-Fei Jian-Pi granules for lung-spleen qi deficiency, Bu-Fei Yi-Shen granules for lung-kidney qi deficiency, and Yi-Qi Zi-Shen granule for lung-kidney qi and yin deficiency.

The TCM granules were compound preparations of TCM and its components are shown in Table 2. These components were produced and packed by Jiang Yin Tian Jiang Pharmaceutical Co. Ltd. with the authentication quality of Goods Manufacturing Practice (Approval Number: SU J0677), Jiangsu, PR China. The test results of drug quality were consistent with the required quality standards.

Bu-Fei Jian-Pi granules (batch number: 080103), came in packs of six bags, and each bag contained 3.83 g. Bu-Fei Yi-Shen granules (batch number: 080102), came in packs of six bags, and each bag contained 4.25 g. Yi-Qi Zi-Shen granules (batch number: 080104), came in packs of six bags, and each bag contained 5.16 g. Each type of granule was given orally, three bags each time, twice a day for 6 months.

Table 2 Main components of traditional Chinese medicine treatment

Chinese name	Latin name	Amount (g)
**Bu-Fei Jian-Pi granules**		
Huang Qi	*Astragalus propinquus*	15
Dang Shen	*Codonopsis pilosula*	15
Bai Zhu	*Atractylodes macrocephala*	12
Fu Ling	*Wolfiporia extensa*	12
Chuan Bei Mu	*Fritillaria cirrhosa*	9
**Bu-Fei Yi-Shen granules**		
Ren Shen	*Radix Ginseng*	9
Huang Qi	*Astragalus propinquus*	15
Gou Qi Zi	*Lycium barbarum*	12
Shan Zhu Yu	*Cornus officinalis*	12
Yin Yang Huo	*Epimedium brevicornu*	9
**Yi-Qi Zi-Shen granules**		
Ren Shen	*Radix Ginseng*	9
Huang Jing	*Polygonatum sibiricum*	15
Shu Di Huang	*Rehmannia glutinosa*	15
Mai Dong	*Ophiopogon japonicus*	15
Wu Wei Zi	*Schisandra chinensis*	9

Outcomes

Frequency and duration of acute exacerbation of COPD

Acute exacerbation of COPD (AECOPD) refers to the patients' acute exacerbation of their original conditions of dyspnea, cough, and (or) expectoration in the development of the disease, which exceeds the daily routine variation, and requires a change in treatment. During the course of AECOPD, patients with aggravation of shortness of breath are often accompanied by dyspnea, chest tightness, a worse cough, increased sputum volume, changes in color, and (or) viscosity of sputum and fever [14].

The frequency and duration of AECOPD each time during treatment were recorded for 6 months and at a follow-up survey for 12 months. If the interval between two onsets of acute exacerbation was within 1week, it was counted as one time of acute exacerbation.

Lung function

The indicators of forced vital capacity (FVC), forced expiratory volume in one second (FEV1) and FEV1 percentage of the predicted value (FEV1%) were tested.

Symptoms

Symptoms included coughing, sputum, shortness of breath, dyspnea, gasping, and cyanosis. Based on the typical format of a Likert response scale [15], each symptom had a score from 0 to 3 according to the severity of the symptom. The lower the score, the better the clinical symptom of the patients. Total scores were got by adding up each symptom score.

Table 1 Therapy at each stage of stable COPD

Classification	Lung function	Therapy
I: Mild	$FEV_1/FVC<0.70$, $FEV_1\geq80\%$ predicted	Active reduction of risk factors; add short-acting bronchodilator (when needed), i.e. albuterol sulfate (inhalation aerosol, Ventolin, GlaxoSmithKline), 100μg/dose, 200 inhalations.
		Dosing: 1–2 inhalations of 100 μg each time, and the maximum dose is 8–12 inhalations a day.
II: Moderate	$FEV_1/FVC<0.70$, $50\%\leq FEV_1<80\%$ predicted	Based on therapy of GOLD 1; add regular treatment with one long-acting bronchodilators,i.e.formoterol fumarate dehydrate (Inhalation powder, Oxis Turbuhaler, AstraZeneca), 4.5μg/dose, 60 inhalations.
		Dosing: one inhalation of 4.5μg each time, twice daily
III:Severe	$FEV_1/FVC<0.70$, $30\%\leq FEV_1<50\%$ predicted	Based on therapy of GOLD 2; add inhaled glucocorticosteroids if repeated exacerbations,i.e. salmeterol/ fluticasone propionate ([dry powder inhaler], Seretide, GlaxoSmithKline), 50/250 μg/dose, 60 inhalations .
		Dosing: one inhalation of 250/50 μg each time, twice daily

The 6-minute walking distance (6MWD)

The 6MWD [16] was measured by the 6-minute walking test (6MWT) which evaluated the distance a person can walk on a flat surface in six minutes.

Dyspnea scale questionnaire

The Dyspnea Scale Questionnaire was firstly developed by the British Medical Research Council [17] (MRC) and later modified by the American Thoracic Society [18], and it contains 5 grades, ranging from 0 to 4.

Quality of life

The World Health Organization of Life-BREF (WHO-QOL-BREF) instrument [19] was adopted, which containes 26 questions in 4 domains, including physical, psychological, society, and environment domains. In each domain, the higher the score of the questionnaire, the better the quality of life of the patients.

Safety

Routine blood, urine, and stool tests, liver and kidney function tests, and an electrocardiogram were performed. Adverse events were recorded at any time during the treatment period and follow-up period.

We recorded the dates when lung function, symptoms, 6MWD, the Dyspnea Scale Questionnaire, and quality of life were observed before treatment (month 0), in the 3rd month (month 3) and 6th month (month 6) during the treatment period, and at the 12th month (month 18) during the follow-up period. The date of AECOPD was observed and calculated at month 0, month 6, and month 18. The dates of safety were measured at months 0 and 6.

Statistical analysis

Statistical analysis set

A full analysis set (FAS) was used to analyze the baseline data and clinical evaluation data of the cases who went through randomization and received treatments, and were observed at least one related record on time point. Partially missing data of the clinical evaluation were carried forward with the principle of the last visit carried forward (LOCF). A per-protocol analysis set (PPS) was used to analyze the clinical evaluation data of the cases who fully completed the trial with better compliance. A safety set (SS) was used to analyze the safety data of the cases who took the trial medicine at least once.

Data processing and statistical analysis methods

All P values were two-tailed and the α level of significance was set at 0.05. Measurement data were described by mean and standard deviation ($\bar{x} \pm SD$) or median. Independent-samples t-tests or Mann–Whitney U-tests were used based on data distribution to compare differences between the two groups. The paired samples t-tests or signed rank sum test was used based on data distribution to compare differences between pre-treatment and post-treatment within one group. Repeated measures were used to compare differences of time continuous observations. Numercial data are described by constituent ratio. The chi-square test was used to compare differences in safety between the trial group and control group. All statistical analyses were undertaken using SAS9.2 (KEY: FQ37-WSB8-7G5C).

Results

Study population

A total of 637 COPD patients were enrolled and assessed for eligibility, through the 2-week washout period, and 352 patients underwent randomization (Figure 1). Based on the withdrawal and rejection criteria, two patients who violated the protocol were excluded. Forty-four patients who did not fully complete the study were withdrawn because of poor compliance, being lost to follow up, or dropped out of the study without explanation. A total of 306 patients fully completed the study, with 155 in the trial group and 151 in the control group. Therefore, the PPS population was 306. The clinical evaluation data of the 44 patients who did not fully complete the study, but received treatment and also completed observation at least one related record on time point were analyzed. These data were evaluated by intent-to-treat analysis according to the principle of the last visit carried forward (LOCF). Therefore, the FAS population was 350, with 175 in the trial group and 175 in the control group.

Demographic and baseline characteristics of the patients are shown in Table 3. There was no significant difference in gender, age, the course of disease, body mass index (BMI), exacerbations, lung function, and GOLD classification of lung function between the two groups.

Comparison of the frequency and duration of acute exacerbation

Before treatment, there was no significant difference in the frequency of acute exacerbation between the two groups (FAS: $P = 0.169$; PPS: $P = 0.129$). For the time point of month 6 and month 18, there was a significant difference in the frequency of acute exacerbation between the trial and control groups (FAS: $P = 0.001$, $P = 0.000$; PPS: $P = 0.004$, $P = 0.000$). The average frequency of acute exacerbation and the constituent ratio of frequency were significantly different between the trial and control groups (FAS: $P = 0.000$, $P = 0.000$; PPS: $P = 0.000$, $P = 0.000$). At 6 and 18 months, the average duration of acute exacerbation was significantly different between the trial and the control groups (FAS: $P = 0.000$; PPS: $P = 0.001$). The results were shown in Table 4.

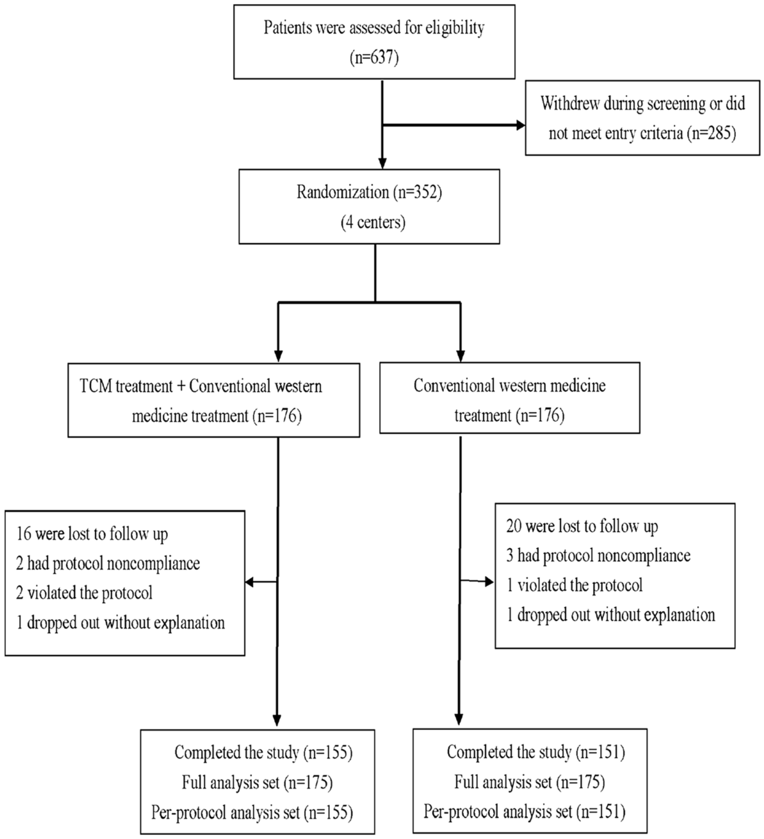

Figure 1 Enrollment of the patients and completion of the study.

Comparison of lung function

FEV1 was significantly higher over time in the trial group, compared with that in the control group (FAS: P = 0.007; PPS: P = 0.008). At 3, 6, and 18 months, FEV1 was significantly higher in the trial group compared with that in the control group (FAS: P = 0.032, P = 0.047, P = 0.001; PPS: P = 0.015, P = 0.021, P = 0.001). There were no significant difference in FVC, FEV1% between the two groups (FAS: P = 0.147, P = 0.204; PPS: P = 0.112, P = 0.126) (Figure 2A,B,C).

Comparison of symptoms and the dyspnea scale

There was no significant difference in total scores of symptoms and the dyspnea scale between the two groups before treatment (symptoms, FAS: P = 0.859; PPS: P = 0.745; dyspnea scale, FAS: P = 0.322; PPS: P = 0.503). The total scores of symptoms and the dyspnea scale of trial group was significantly lower over time compared with those of the control group (FAS: P = 0.001, PPS: P = 0.001; FAS: P = 0.002; PPS: P = 0.004). At 3, 6 and 18 months, there was significant difference in total scores of symptoms and the dyspnea scale in the trial group compared with those in the control group (symptoms, FAS: P = 0.000, P = 0.000, P = 0.000; PPS: P = 0.000, P = 0.000, P = 0.001; dyspnea scale, FAS: P = 0.008, P = 0.000, P = 0.000; PPS: P = 0.014, P = 0.001, P = 0.000) (Figure 2D, F).

Comparison of 6MWD

There was no significant difference in the mean value of 6MWD between two groups before treatment and month 3 (FAS: P = 0.986, P = 0.05; PPS: P = 0.781, P = 0.064). The mean value of 6MWD in the trial group was significantly higher over time than that of the control group (FAS: P = 0.045; PPS: P = 0.042). At 6 and 18 months, the mean value of 6MWD were significantly higher in trial group compared with the control group (FAS: P = 0.004, P = 0.009; PPS: P = 0.003, P = 0.009) (Figure 2E).

Comparison of quality of life

Before treatment, there was no significant difference in the quality of life scores in each domain between the groups (physical domain, FAS: P = 0.885; PPS: P = 0.943; psychological domain, FAS: P = 0.884; PPS: P = 0.723; social domain, P = 0.979; PPS: P = 0.884; environment domain, FAS: P = 0.763, PPS: P = 0.107). Quality of life scores of the trial group continued to increase over time, and the mean scores of the trial group were significantly higher than those of the control group (physical domain, FAS: P = 0.000, PPS:

Table 3 Baseline characteristics of the patients

Characteristics	Full analysis set				Per-protocol analysis set			
	Trial n=175	Control n=175	$t/\chi^2/Z$	P	Trial n=155	Control n=151	$t/\chi^2/Z$	P
Age (years)	66.33 ± 9.63	64.28 ± 9.42	−0.935	0.351	62.74 ± 9.87	64.66 ± 8.92	−1.778	0.076
Course of disease△	169.56 ± 290.63	161.07 ± 128.45	0.353	0.725	166.86 ± 305.92	163.18 ± 131.84	0.136	0.892
BMI▲	24.26 ± 11.79	23.75 ± 3.10	0.543	0.587	24.41 ± 12.54	23.74 ± 3.09	0.643	0.521
Exacerbation□								
Frequency (times)	3.26 ± 2.27	2.94 ± 2.05	1.378	0.169	3.32 ± 2.31	2.95 ± 1.91	1.523	0.129
Duration (days)	2.78 ± 2.00	2.73 ± 1.97	0.228	0.820	2.82 ± 2.08	2.75 ± 1.81	0.319	0.750
Lung function■								
FVC (liters)	2.91 ± 0.95	2.81 ± 0.89	0.953	0.341	2.95 ± 0.97	2.81 ± 0.92	1.239	0.216
FEV1(liters)	1.46 ± 0.57	1.35 ± 0.46	1.465	0.144	1.46 ± 0.57	1.35 ± 0.46	1.747	0.082
FEV1%	49.89 ± 10.84	49.58 ± 12.07	0.244	0.807	49.94 ± 10.95	49.58 ± 12.36	0.266	0.790
Gender								
Male	122	131	1.556	0.212	106	116	2.732	0.098
Female	54	43			49	35		
Smoking status								
Currently smoking	103	114	1.817	0.178	89	102	3.346	0.067
None-smoking	73	60			66	49		
Smoking pack-years	380.91 ± 158.26	366.47 ± 150.32	0.686	0.493	377.08 ± 164.08	372.47 ± 154.47	0.199	0.843
GOLD classification								
GOLD 1	14	6	−0.093	0.926	13	6	−0.369	0.712
GOLD 2	68	80			61	66		
GOLD 3	94	88			81	79		

△The course of disease was calculated in months.
▲The body-mass index (BMI)is the weight in kilograms divided by the square of the height in meters.
□Exacerbations during the 12 months before screening were self-reported.
■Clinical data are from visit 1 (the screening visit). FEV1 denotes forced expiratory volume in 1 second, and FVC is forced vital capacity.

P = 0.000; psychological domain, FAS: P = 0.000, PPS: P = 0.000; social domain, FAS: P = 0.000, PPS: P = 0.000; environment domain, FAS: P = 0.000, PPS: P = 0.000). At 3, 6, 18 months, the trial group had higher quality of life scores compared with those of the control group (physical domain, FAS: P = 0.022, P = 0.000, P = 0.001; PPS: P = 0.018, P = 0.000, P = 0.001; psychological domain, FAS: P = 0.049, P = 0.001, P = 0.003; PPS: P = 0.045, P = 0.001, P = 0.003; social domain, FAS: P = 0.016, P = 0.000, P = 0.000; PPS: P = 0.006, P = 0.000, P = 0.000; environment domain, FAS: P = 0.044, P = 0.001, P = 0.006; PPS: P = 0.045, P = 0.001, P = 0.006) (Figure 3A, B, C, D).

Table 4 Comparison of the frequency and duration of acute exacerbation

Variable	Full analysis set				Per-protocol analysis set			
	Trial n=175	Control n=175	t/Z	P	Trial n=155	Control n=151	t/Z	P
Frequency (times)								
Month 0	3.26 ± 2.27	2.94 ± 2.05	1.378	0.169	3.32 ± 2.31	2.95 ± 1.91	1.523	0.129
Month 6	1.02 ± 1.51	1.71 ± 2.14	−3.484	0.001	0.97 ± 1.26	1.48 ± 1.81	−2.888	0.004
Month 18	0.49 ± 0.77	1.09 ± 1.15	−5.689	0.000	0.54 ± 0.75	1.01 ± 0.98	−4.798	0.000
Average frequency	1.01 ± 1.26	1.95 ± 1.88	−5.509	0.000	1.02 ± 1.08	1.76 ± 1.47	−4.963	0.000
Frequency (constituent ratio)								
Have exacerbation	101	136	18.5857	0.000	92	121	15.6084	0.000
None exacerbation	76	37			63	30		
Duration(days)								
Average duration	4.20 ± 5.20	6.22 ± 5.15	−3.653	0.000	4.39 ± 5.24	6.37 ± 5.19	−3.323	0.001

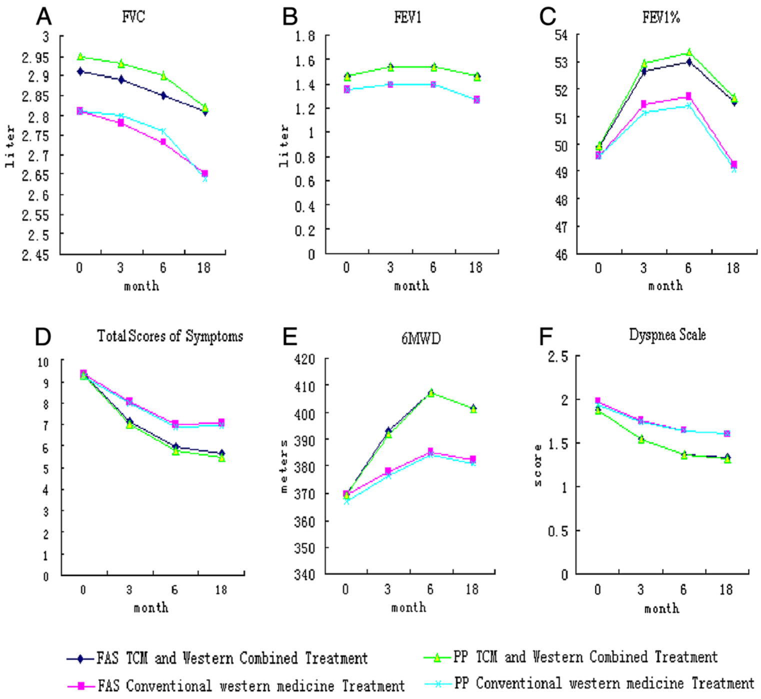

Figure 2 Comparison of the results of FVC, FEV1, FEV1%, Total Scores of symptoms, 6MWD and Dyspnea Scale.

Evaluation of safety

There were no significant differences in routine blood, urine and stool tests, liver and kidney function tests, and electrocardiogram in each group before and after treatment. There were no serious adverse events during the treatment and follow-up period of the trial. However, adverse events were recorded during treatment and follow-up, but there was no significant difference in adverse events between the two groups ($P > 0.05$). In the trial group, five cases had adverse events that may have been caused by drug-related effects of TCM treatment combined with conventional Western medicine. Among these five cases, one case had abdominal distension, one case had palpitation, one case had constipation, one case had thirst, and one case had insomnia. In the control group, eight cases had adverse events that may have been caused by drug-related effects of conventional Western medicine. Among these eight cases, one case had abdominal distension, one case had palpitation, one cases had constipation, one case had thirst, one case had insomnia, and one cases had stomach discomfort, one cases had dry throat.

Discussions

COPD is a common and deadly disease that is characterized by persistent and progressive airflow limitation. Therefore, exacerbations and comorbidities contribute to overall severity in individual patients. Presently, the classes of medications recommended by GOLD are commonly used in treating COPD. Although more alternative approaches are used in COPD patients, definite effect evidence for TCM treatment is still limited. Hence, based on conventional Western medicine that referred to the GOLD classes of medications, we conducted our study to evaluate the efficacy and safety of TCM treatment by measuring such outcomes, scuh as exacerbations, lung function, symptoms, exercise tolerance and quality of life.

The TCM treatment in this study included Bu-Fei Jian-Pi granules, Bu-Fei Yi-Shen granules and Yi-Qi Zi-Shen granules. These granules are made from highly concentrated, selected Chinese herbs, and are produced in accordance with a traditional Chinese formula. The choice within each granule depends on the patient-specific pattern on which based the TCM theory of lung, spleen and kidney and Chinese medicine pattern theory. Over the 6-month treatment and 12-month follow-up, the TCM granules had beneficial effects on the measured outcomes, with no relevant between-group differences in adverse events.

AECOPD and lung function were the primary outcomes in this study. AECOPD can affect a decrease in lung function and impair the health-related quality of life [20]. It has been reported that the in patients admitted to intensive care units for AECOPD, their mortality rate was about 11–24% [21,22]. Therefore, the reductions in the frequency and duration of exacerbations are a major goal of COPD management and an important

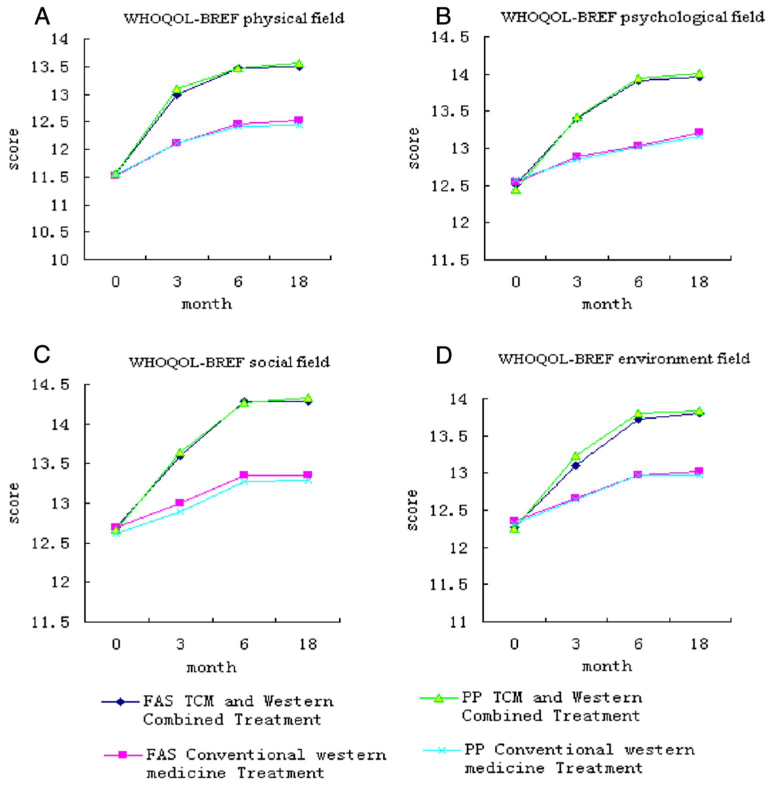

Figure 3 Comparison of the results of WHOQOL-BREF physical field, psychological field, social field, and environment field.

indicator for evaluating the treatment [23]. An accelerated decline in lung function is the main hallmark of COPD. A previous study showed that the rate of decline in FVE1 in COPD patients decreased from 47 ml to 69 ml per year [24]. According to the results from the well-known UPLIFT Trial and TORCH Trial, the rate of decline in FVE1 in the salmeterol/fluticasone group was 39 ml/yr and the rate of decline in the tiotropium group was 41 ml/yr [4,25]. Our results showed that the frequency and the duration of AECOPD in the trial group was 1.02 times and 4.39 days, respectively; however, those in the control group were 1.76 times and 6.37 days respectively. The rate of decline in FVE1 in patients in the trial group increased by approximately 4.16 ml per year, and it decreased approximately 52.54 ml/yr in the control group. The rate of decline in FVE1 in patients in the control group was consistent with that of previous large trials. TCM granules can reduce the frequency and duration of acute exacerbation, and maintain the rate of decline in FEV1. This may be due to the combined effect of conventional Western medicine and Chinese medicines.

Symptoms, exercise tolerance and health status outcomes were also observed in the current study. The characteristic symptoms of COPD are chronic and progressive dyspnea, cough, and sputum production. Although COPD is defined on the basis of airflow limitation, in practice the decision to seek medical help is usually determined by the effect of a symptom on a patient's daily life. However, in an early or stable state, dyspnea is not obvious and often overlooked by patients and physicians [26]. Evaluation of the degree in breathing difficulty is important to understand the severity of COPD, the health status of patients and to evaluate clinical intervention effects. Therefore, observations of symptoms and the Dyspnea Scale questionnaire were used in the current study [27]. The 6MWD is widely used in respiratory disease as a comprehensive evaluation of functional status of the body with moderate or severe disease [28]. The main objective of the 6MWD is to determine exercise tolerance and oxygen saturation during submaximal exercise. The 6MWD is also considered useful in the determination of the point at which patients should be listed for rehabilitation programs. The cut-off point for the 6MWD was approximately 350 m [29] and the minimal important difference in COPD patients is 25 m [30]. Currently, quality of life is an indispensable indicator and assessment tool. Because of the long course of COPD and a progressive decline in lung function, patients' daily activities are limited and they rely on on family members more, coupled with a financial burden and decreased family status. COPD patients usually

have low self-esteem, depression, anxiety, and other adverse effects on psychological emotion and social adaptation ability. There are many types of effective and reliable health related quality of life questionnaires on chronic respiratory diseases [31]. With better reliability and validity, the WHOQOL-BREF was adopted in the current study to evaluate the quality of life of patients with COPD. Our results showed that the improvement of 6MWD in the trial group was 31.84 m, which, was better than that 14.07 m in the control group. The improvement of quality of life was 13.26% in the trial group and 6.13% in the control group, and symptoms were reduced, with lower dyspnea scale scores in the trial group compared with those in the control group. TCM granules reduced symptoms, and improved exercise tolerance and health status.

In addition, the reasons for the favorable effects of TCM treatment on the clinical parameters in COPD patients were also taken into account. Our results suggest that these favorable effects may be due to the combined effect of conventional Western medicine and Chinese medicines. The clinical effect of the TCM granules lied in reducing the frequency of acute exacerbation, ameliorating symptoms, increasing exercise endurance, and improving quality of life. In view of our previous studies, the mechanism of the TCM granules may be involved in reducing the expression of interleukin (IL)-8, IL-6, IL-10, IL-1β and TNF-α, and regulating the level of inflammatory cytokines. TCM granules may also be involved in regulating the level of cellular immunity, especially the expression of T lymphocyte subsets (CD3+, CD4+, CD8+, CD4+/CD8+) and CD4+CD25+; reducing the expression of matrix metalloproteinase(MMP)-2, MMP-9, improving the expression of tissue inhibitor of metalloproteinases-1, regulating the balance of MMPs, and reducing the expression of JAK/STAT signaling pathways related factors and inflammatory factors.

Based on the the results of our trial, TCM treatment, including Bu-Fei Jian-Pi granules, Bu-Fei Yi-Shen granules, and Yi-Qi Zi-Shen granules, is safe and effective for treating COPD patients. However, there are some limitations for this study. In this study, herbal interventions (the granules) for the three TCM patterns were recognized as whole comprehensive interventions rather than one herbal intervention for each pattern. This study aimed to evaluate comprehensive interventions based on the three common patterns in stable COPD patients. Therefore, the efficacy of each type of herbal intervention on a TCM pattern was not evaluated. In addition, the 6-month treatment duration and 12-month follow-up duration was a little short to observe changes in lung function and to show the fully effect of TCM treatment. Further studies should be performed to evaluate TCM treatment.

Conclusions

Based on the TCM patterns, TCM treatments for COPD are safe and effective. The curative effects of TCM were mainly manifested by reducing the frequency and duration of acute exacerbation, ameliorating symptoms, and improving exercise endurance, and quality of life. Further studies are required to prove the efficacy and safety of TCM treatments

Abbreviations

COPD: Chronic obstructive pulmonary disease; TCM: Traditional Chinese medicine; AECOPD: Acute exacerbation of COPD; FVC: Forced vital capacity; FEV1: Forced expiratory volume in one second; FEV1%: FEV1 percentage of predicted value; 6MWD: The six-minute walking distance; QOL: Quality of life.

Competing interests

The authors declare that they have no competing interests.

Authors' contributions

This project was initiated and developed by LJS and LSY. LJS and LSY were involved in the design of the study and the interventions of the protocol. WMH and XY were involved in drafting and writing the manuscript. YXQ was involved in evaluating the data. SZK, MLJ and ZW were involved in coordinating the study and supervising the work. ZHL, CF, and PYC were involved in performing the study. All authors read and approved the final manuscripts.

Acknowledgments

This study was supported jointly by the National Key Technology R&D Program during the 11th Five-Year Plan Period (2006BA104A13-02) and the Program for New Century Excellent Talents at the University of Henan Province (2006HANCET-05).
The authors are grateful to The Ministry of Science and Technology of the People's Republic of China and the State Administration of Traditional Chinese Medicine of the People's Republic of for funding this study.

Author details

[1]Department of Respiratory Diseases, The First Affiliated Hospital of Henan University of Traditional Chinese Medicine, No. 19 Renmin Road, Zhengzhou, PR China. [2]The Geriatric Department, Henan University of Traditional Chinese Medicine, No. 1 Jinshui Road, Zhengzhou, PR China. [3]Department of Respiratory Diseases, Jiangsu Provincial Hospital of Traditional Chinese Medicine, No. 155 Hanzhong Road, Nanjing, PR China. [4]Department of Respiratory Diseases, Henan Provincial People's Hospital, No. 5 Weiwu Road, Zhengzhou, PR China. [5]Department of Respiratory Diseases, Affiliated Hospital of Shandong University of Traditional Chinese Medicine, No. 42 Wenhua West Road, Jinan, PR China.

Received: 16 January 2012 Accepted: 24 October 2012
Published: 29 October 2012

References

1. Raherison C, Girodet PO: **Epidemiology of COPD.** *Eur Respir Rev* 2009, **18**:213–221.
2. Lin HH, Murray M, Cohen T, Colijn C, Ezzati M: **Effects of smoking and solid-fuel use on COPD, lung cancer, and tuberculosis in China: a time-based, multiple risk factor, modelling study.** *Lancet* 2008, **372**(9648):1473–1483.
3. Qaseem A, Wilt TJ, Weinberger SE, Hanania NA, Criner G, van der Molen T, Marciniuk DD, Denberg T, Schünemann H, Wedzicha W, MacDonald R, Shekelle P: **Diagnosis and management of stable chronic obstructive pulmonary disease: a clinical practice guideline update from the American College of Physicians, American College of Chest Physicians, American Thoracic Society, and European Respiratory Society.** *Ann Intern Med* 2011, **155**(3):179–191.
4. Calverley PM, Anderson JA, Celli B, Ferguson GT, Jenkins C, Jones PW, Yates JC, Vestbo J: **Satmeterol and flutieasene propionate and survival in chronic obstructive pulmonary disease.** *N Engl J Med* 2007, **356**(8):775–789.

5. Li JS, Wang ZW, Yu XQ, Wang MH, Li SY: **Clinical efficacy and safety of TCM for COPD at stable phase: A Systematic review.** *Liaoning Zhong Yi Za Zhi* 2010, **37**(2):229–232.
6. Li JS, Wang ZW, Yu XQ, Wang MH, Li SY: **Systemic evaluation of TCM for COPD in the acute stage of exacerbation.** *Tianjin Zhong Yi Yao* 2008, **25**(5):428–431.
7. Zhang WJ, Zhang YP: **Recent research in chronic obstructive pulmonary disease treated with TCM.** *Beijing Zhong Yi Yao Da Xue Xue Bao (Clinical Medicine)* 2007, **14**(5):39–41.
8. Lu AP, Chen KJ: **Chinese medicine pattern diagnosis could lead to innovation in medical sciences.** *Zhong Guo Zhong Xi Yi Jie He Za Zhi* 2011, **17**(11):811–817.
9. Wang ZW, Li JS, Yu XQ, Wang MH, Li SY: **Symptom diagnosis criteria for chronic obstructive pulmonary diseases at stationary phase from literature.** *Zhong Yi Yan Jiu* 2008, **21**(8):55–58.
10. Global Initiative for Chronic Obstructive Lung Disease: *Global strategy for the diagnosis, management, and prevention of chronic obstructive pulmonary disease*; *Revised 2006*, http://goldcopd.com/index.2007-01-22.
11. COPD Study Group of Chinese Society of Respiratory Disease: **Treatment guidelines of chronic obstructive pulmonary disease (revised 2007).** *Zhong Hua Jie He Yu Hu Xi Za Zhi* 2007, **30**(1):8–17.
12. Li JS, Li SY, Wang ZW, Yu XQ, Wang MH, Wang YY, The internal medicine branch of China Association of Chinese Medicine: **Syndrome diagnostic criteria of TCM of Chronic Obstructive Pulmonary Disease (2011 Edition).** *Zhong Yi Za Zhi* 2012, **53**(2):177–178.
13. Hong ML, Chen WX, Cai SH, Gao LY, Dai SZ, Chen ZB: **The interventional effects of yufeining on lung function in patients with chronic obstructive pulmonary disease.** *Zhong Hua Zhong Yi Yao Za Zhi* 2005, **20**(2):92–95.
14. Celli BR, MacNee W: **Standards for the diagnosis and treatment of patients with COPD: a summary the ATS/ERS position paper.** *Eur Respir J* 2004, **23**(6):932–946.
15. Jamieson S: **Likert scales: how to (ab)use them.** *Med Educ* 2004, **38**(12):1217–1218.
16. ATS: **ATS statement: guidelines for the Six-minute walk test.** *Am J Respir Crit Care Med* 2002, **166**:111–117.
17. Fletcher CM, Elmes PC, Fairbairn MB, Wood CH: **The significance of respiratory symptoms and the diagnosis of chronic bronchitis in a working population.** *Br Med J* 1959, **2**(5147):257–266.
18. Mahler DA, Well CK: **Evaluation of clinical methods for rating dyspnea.** *Chest* 1988, **93**(3):580–586.
19. WHO: *The world health organization quality of life (WHOQOL-BREF)*. Geneva: World Health Organization; 2004.
20. Bourbeau J, Ford G, Zackon H, Pinsky N, Lee J, Ruberto G: **Impact on patients' health status following early identification of a COPD exacerbation.** *Eur Respir J* 2007, **30**(5):907–913.
21. Burge S, Wedzicha JA: **COPD exacerbations: definitions and classifications.** *Eur Respir J* 2003, **21**(41):46–53.
22. Wedzicha JA, Seemungal TA: **COPD exacerbations: defining their cause and prevention.** *Lancet* 2007, **370**(9589):786–796.
23. Li JS, Wang MH: **The clinical significance of acute exacerbation of chronic obstructive pulmonary disease.** *Zhong Guo Wei Zhong Bing Ji Jiu Yi Xue* 2007, **19**(9):572–573.
24. Lung Health Study Research Group: **Effect of inhaled triamcinolone on the decline in pulmonary function in chronic obstructive pulmonary disease.** *N Engl J Med* 2000, **343**(26):1902–1909.
25. Tashkin DP, Celli B, Senn S, Burkhart D, Kesten S, Menjoge S, Decramer M: **A 4-year trial of tiotropium in chronic obstructive pulmonary disease.** *N Engl J Med* 2008, **359**(15):1543–1554.
26. Li JS, Li SY, Wang MH: **Appraisal investigating progression of chronic obstructive pulmonary dyspnea.** *Henan Zhong Yi Xue Yuan Xue Bao* 2007, **22**(129):79–80.
27. Camargo LA, Pereira CA: **Dyspnea in COPD: beyond the modified medical research council scale.** *J Bras Pneumol* 2010, **36**(5):571–578.
28. Morales-Blanhir JE, Palafox Vidal CD, Rosas Romero Mde J, García Castro MM, Londoño Villegas A, Zamboni M: **Six-minute walk test: a valuable tool for assessing pulmonary impairment.** *J Bras Pneumol* 2011, **37**(1):110–117.
29. Celli BR, Cote CG, Marin JM, Casanova C, Montes de Oca M, Mendez RA, Pinto Plata V, Cabral HJ: **The body-mass index, airflow obstruction, dyspnea, and exercise capacity index in chronic obstructive pulmonary disease.** *N Engl J Med* 2004, **350**(10):1005–1012.
30. Holland AE, Hill CJ, Rasekaba T, Lee A, Naughton MT, McDonald CF: **Updating the minimal important difference for six-minute walk distance in patients with chronic obstructive pulmonary disease.** *Arch Phys Med Rehabil* 2010, **91**(2):221–225.
31. Li JS, Li SY, Wang MH: **Functional status and living quality change of chronic obstructive pulmonary diseases.** *Henan Zhong Yi Xue Yuan Xue Bao* 2007, **22**(1):19–22.

doi:10.1186/1472-6882-12-197
Cite this article as: Li *et al.*: Effects of comprehensive therapy based on traditional Chinese medicine patterns in stable chronic obstructive pulmonary disease: a four-center, open-label, randomized, controlled study. *BMC Complementary and Alternative Medicine* 2012 **12**:197.

Submit your next manuscript to BioMed Central and take full advantage of:

- **Convenient online submission**
- **Thorough peer review**
- **No space constraints or color figure charges**
- **Immediate publication on acceptance**
- **Inclusion in PubMed, CAS, Scopus and Google Scholar**
- **Research which is freely available for redistribution**

Submit your manuscript at
www.biomedcentral.com/submit

BioMed Central
