# Supplementary material for: The Effects of Traditional Chinese Exercise in Patients with Chronic Obstructive Pulmonary Disease: A Meta-Analysis
Source: PLoS One. 2016 Sep 2;11(9):e0161564. doi: 10.1371/journal.pone.0161564 (PMC5010221; doi:10.1371/journal.pone.0161564)
Supplement: S2 Appendix — (ZIP) [file pone.0161564.s002.zip › S2 Appendix Reasons for excluded articles/Article4 .pdf]

Eur Respir J 2013; 41: 1005–1007
DOI: 10.1183/09031936.00128512
Copyright©ERS 2013

EDITORIAL

T'ai chi for individuals with COPD: an ancient wisdom for a 21st century disease?

Martijn A. Spruit* and Michael I. Polkey#

Even when receiving optimal medical treatment, individuals with chronic obstructive pulmonary disease (COPD) commonly experience daily symptoms of dyspnoea and fatigue [1, 2]. Consequently exercise intolerance may render activities of daily life problematic, leading to a downward spiral in which there is a progressive reduction in physical activity [3–8]. Thus, individuals with COPD can end up in a vicious circle of physical deconditioning, including loss of lower limb muscle mass and strength, loss of balance and osteoporosis [8–11].

In the past 20 years, pulmonary rehabilitation has become an integrated part of the management of individuals with moderate to very severe COPD [12, 13]. Pulmonary rehabilitation, defined as an evidence-based, multidisciplinary and comprehensive intervention for patients with chronic respiratory diseases who are symptomatic and often have decreased daily life activities, is designed to reduce symptoms, optimise functional status and increase participation in activities of daily living [14]. Pulmonary rehabilitation, which usually involves an initial assessment, exercise training, education and behaviour change, is safe and effective in individuals with COPD [14–16]. Indeed, improvements in health-related quality of life, functional mobility and lower limb muscle function have been reported [17]. Exercise training is the cornerstone of pulmonary rehabilitation and mostly consists of aerobic exercises (stationary cycling, treadmill walking and/or ground walking) in combination with muscle strengthening exercises (resistance training and/or transcutaneous neuromuscular electrical stimulation) [14, 15, 18, 19].

T'ai chi is a traditional Chinese form of conditioning exercise derived from martial arts. When practised correctly, t'ai chi is thought to strengthen the body's vital energy and enhance the passage of this energy throughout the body to confer its health-promoting effects. T'ai chi has spread worldwide as improvements in physical and emotional functioning have frequently been reported in older adults [20], as well as in individuals with fibromyalgia [21], Parkinson's disease [22] or knee osteoarthritis [23]. Sun-style t'ai chi was developed by Sun Lutang and it is well known for its smooth, flowing movements [24]. The footwork of Sun-style t'ai chi is unique, when one foot advances or retreats the other follows. It also uses an open palm throughout the entirety of its main form and exhibits small circular movements with the hand. Its gentle postures and high stances may make it very suitable for individuals with COPD. In this issue of the *European Respiratory Journal*, LEUNG *et al*. [25] present new insights into the effects of Sun-style t'ai chi in individuals with COPD. 42 individuals with COPD were randomly assigned to a usual care control group or a twice weekly supervised Sun-style t'ai chi training in combination with an unsupervised, home-based t'ai chi training programme (including a t'ai chi training booklet and DVD) on the remaining 5 days a week (30 min per day) for a total duration of 12 weeks. Improvements in lower limb muscle function, balance, functional exercise performance, health-related quality of life and mood status following Sun-style t'ai chi were significantly better compared to the usual care control group. Indeed, the mean differences between groups in changes in incremental shuttle walk distance, endurance shuttle walk time, and health-related quality of life questionnaire scores exceeded the minimal important difference of 47.5 m, 186 s or 0.5 points per question, respectively [26–28]. Interestingly, individuals with COPD used on average 63% of their peak aerobic capacity during a session of Sun-style t'ai chi, which is similar to the mean oxygen uptake during a session of lower limb resistance training [29, 30].

Where then does this leave t'ai chi as compared with conventional exercise training? We believe this question could be answered from two directions. The classical western scientific approach would be to hypothesise that t'ai chi was superior to standard exercise training (or *vice versa*). Then having obtained the necessary pilot data in order to calculate the required sample size a prospective randomised controlled study would be undertaken to assess the effect of both therapies on a chosen primary end-point at a single time point, with the hope that they would differ by more than a pre-specified minimally clinically important difference. However, a hypothesis driven approach, while wholly appropriate for drug studies, may be a poor way of recommending therapy for individuals in an area where active participation is required. This approach may be considered as a way to address the relative merits of t'ai chi and exercise training in a second dimension. Specifically, we postulate that the benefits of

*Program Development Centre, CIRO+, Centre of Expertise for Chronic Organ Failure, Horn, The Netherlands. #NIHR Respiratory Biomedical Research Unit, Royal Brompton Hospital and Imperial College, London, UK.

CORRESPONDENCE: M.A. Spruit, Program Development Centre, CIRO+, Centre of Expertise for Chronic Organ Failure, Hornerheide 1, 6085 NM, Horn, The Netherlands. E-mail: martijnspruit@ciro-horn.nl

interventions which aim to increase physical activity are conferred by taking part. Therefore, uptake and adherence are likely to greatly outweigh technical differences between interventions.

In this respect t'ai chi may offer a number of attractions compared with conventional exercise-based pulmonary rehabilitation and may therefore facilitate exercise training for some individuals with COPD. First, considered globally, only a small proportion of individuals with COPD receive pulmonary rehabilitation [31] in part due to funding or reimbursement issues [32]; t'ai chi might very well be cheaper, particularly in countries with a large practitioner base. Secondly, conventional exercise training requires gym equipment and space in which to house them, which entails the creation of a central location; travel distance to the pulmonary rehabilitation facility has been identified as a barrier to participation [33, 34]. In contrast, t'ai chi requires no equipment and minimal space. Thirdly, since the positive effects of pulmonary rehabilitation tend to diminish over time one would expect them to be extended by continued exercise [18, 35–38]. Because it requires no apparatus, t'ai chi may be more attractive for maintenance therapy, after an original course of either t'ai chi or standard exercise-based pulmonary rehabilitation, than continued western-style exercise.

Naturally, the data of LEUNG *et al*. [25] have some limitations, although two other studies with a similar design are encouraging [39, 40]. First, the participants in this study were carefully selected; specifically, only 42 of the 139 screened for eligibility were randomised, as a majority of the individuals did not fulfil the inclusion criteria. Secondly, oxygen-dependent individuals were ineligible to participate in this study, and the conclusions should not be uncritically applied to this subset. Thirdly, other exercise modalities which require no exercise apparatus, like Nordic walking and ground walking, have also been shown to improve exercise performance in individuals with COPD [41, 42]. Finally, interdisciplinary pulmonary rehabilitation programmes can address specific issues, such as nutritional modulation, collaborative self-management to promote self-efficacy, advance care planning, and/or smoking cessation, which are not addressed during a course of t'ai chi.

To conclude, 12 weeks of Sun-style t'ai chi improves exercise performance and health-related quality life in individuals with COPD as compared to a usual care control group. A direct comparison between t'ai chi and conventional supervised exercise modalities (*e.g*. cycling, walking and/or strengthening exercises) seems a logical next step in this emerging area of research.

STATEMENT OF INTEREST

Conflict of interest information can be found alongside the online version of this article at www.erj.ersjournals.com

REFERENCES

1 Kessler R, Partridge MR, Miravitlles M, *et al.* Symptom variability in patients with severe COPD: a pan-European cross-sectional study. *Eur Respir J* 2011; 37: 264–272.
2 Janssen DJ, Spruit MA, Wouters EF, *et al.* Daily symptom burden in end-stage chronic organ failure: a systematic review. *Palliat Med* 2008; 22: 938–948.
3 Annegarn J, Meijer K, Passos VL, *et al.* Problematic activities of daily life are weakly associated with clinical characteristics in COPD. *J Am Med Dir Assoc* 2012; 13: 284–290.
4 Vaes AW, Wouters EF, Franssen FM, *et al.* Task-related oxygen uptake during domestic activities of daily life in patients with COPD and healthy elderly subjects. *Chest* 2011; 140: 970–979.
5 Waschki B, Spruit MA, Watz H, *et al.* Physical activity monitoring in COPD: compliance and associations with clinical characteristics in a multicenter study. *Respir Med* 2012; 106: 522–530.
6 Watz H, Waschki B, Meyer T, *et al.* Physical activity in patients with COPD. *Eur Respir J* 2009; 33: 262–272.
7 Shrikrishna D, Patel M, Tanner RJ, *et al.* Quadriceps wasting and physical inactivity in patients with COPD. *Eur Respir J* 2012; 40: 1115–1122.
8 Polkey MI, Moxham J. Attacking the disease spiral in chronic obstructive pulmonary disease. *Clin Med* 2006; 6: 190–196.
9 Graat-Verboom L, van den Borne BE, Smeenk FW, *et al.* Osteoporosis in COPD outpatients based on bone mineral density and vertebral fractures. *J Bone Miner Res* 2011; 26: 561–568.
10 Seymour JM, Spruit MA, Hopkinson NS, *et al.* The prevalence of quadriceps weakness in COPD and the relationship with disease severity. *Eur Respir J* 2010; 36: 81–88.
11 Roig M, Eng JJ, MacIntyre DL, *et al.* Falls in people with chronic obstructive pulmonary disease: an observational cohort study. *Respir Med* 2011; 105: 461–469.
12 Spruit MA, Vanderhoven-Augustin I, Janssen PP, *et al.* Integration of pulmonary rehabilitation in COPD. *Lancet* 2008; 371: 12–13.
13 Wagg K. Unravelling self-management for COPD: what next? *Chron Respir Dis* 2012; 9: 5–7.
14 Nici L, Donner C, Wouters E, *et al.* American Thoracic Society/European Respiratory Society statement on pulmonary rehabilitation. *Am J Respir Crit Care Med* 2006; 173: 1390–1413.
15 Ries AL, Bauldoff GS, Carlin BW, *et al.* Pulmonary rehabilitation: joint ACCP/AACVPR evidence-based clinical practice guidelines. *Chest* 2007; 131: 4S–42S.
16 Lacasse Y, Goldstein R, Lasserson TJ, *et al*. Pulmonary rehabilitation for chronic obstructive pulmonary disease. *Cochrane Database Syst Rev* 2006; CD003793.
17 Spruit MA, Wouters EF. New modalities of pulmonary rehabilitation in patients with chronic obstructive pulmonary disease. *Sports Med* 2007; 37: 501–518.
18 Spruit MA, Troosters T, Trappenburg JC, *et al.* Exercise training during rehabilitation of patients with COPD: a current perspective. *Patient Educ Couns* 2004; 52: 243–248.
19 Sillen MJ, Speksnijder CM, Eterman RM, *et al.* Effects of neuromuscular electrical stimulation of muscles of ambulation in patients with chronic heart failure or COPD: a systematic review of the English-language literature. *Chest* 2009; 136: 44–61.
20 Blake H, Hawley H. Effects of Tai Chi exercise on physical and psychological health of older people. *Curr Aging Sci* 2012; 5: 19–27.
21 Wang C, Schmid CH, Rones R, *et al.* A randomized trial of tai chi for fibromyalgia. *N Engl J Med* 2010; 363: 743–754.
22 Li F, Harmer P, Fitzgerald K, *et al.* Tai chi and postural stability in patients with Parkinson's disease. *N Engl J Med* 2012; 366: 511–519.
23 Hochberg MC, Altman RD, April KT, *et al.* American College of Rheumatology 2012 recommendations for the use of nonpharmacologic and pharmacologic therapies in osteoarthritis of the hand, hip, and knee. *Arthritis Care Res (Hoboken)* 2012; 64: 465–474.
24 Wikipedia. Sun-style t'ai chi ch'uan. http://en.wikipedia.org/wiki/Sun-style_t%27ai_chi_ch%27uan Date last accessed: August 15, 2012. Date last updated: February 24, 2013.
25 Leung RWM, McKeough ZJ, Peters MJ, *et al.* Short-form Sun-style t'ai chi as an exercise training modality in people with COPD. *Eur Respir J* 2013; 41: 1051–1057.
26 Pepin V, Laviolette L, Brouillard C, *et al.* Significance of changes in endurance shuttle walking performance. *Thorax* 2011; 66: 115–120.

27 Singh SJ, Jones PW, Evans R, *et al.* Minimum clinically important improvement for the incremental shuttle walking test. *Thorax* 2008; 63: 775–777.

28 Jaeschke R, Singer J, Guyatt GH. Measurement of health status. Ascertaining the minimal clinically important difference. *Control Clin Trials* 1989; 10: 407–415.

29 Probst VS, Troosters T, Pitta F, *et al.* Cardiopulmonary stress during exercise training in patients with COPD. *Eur Respir J* 2006; 27: 1110–1118.

30 Sillen MJ, Janssen PP, Akkermans MA, *et al.* The metabolic response during resistance training and neuromuscular electrical stimulation (NMES) in patients with COPD, a pilot study. *Respir Med* 2008; 102: 786–789.

31 Brooks D, Sottana R, Bell B, *et al.* Characterization of pulmonary rehabilitation programs in Canada in 2005. *Can Respir J* 2007; 14: 87–92.

32 Yohannes A, Stone R, Lowe D, *et al.* Pulmonary rehabilitation in the United Kingdom. *Chron Respir Dis* 2011; 8: 193–199.

33 Johnston CL, Maxwell LJ, Maguire GP, *et al.* How prepared are rural and remote health care practitioners to provide evidence-based management for people with chronic lung disease? *Aust J Rural Health* 2012; 20: 200–207.

34 Keating A, Lee A, Holland AE. What prevents people with chronic obstructive pulmonary disease from attending pulmonary rehabilitation? A systematic review. *Chron Respir Dis* 2011; 8: 89–99.

35 O'Neill B, McKevitt A, Rafferty S, *et al.* A comparison of twice-*versus* once-weekly supervision during pulmonary rehabilitation in chronic obstructive pulmonary disease. *Arch Phys Med Rehabil* 2007; 88: 167–172.

36 Waterhouse JC, Walters SJ, Oluboyede Y, *et al*. A randomised 2×2 trial of community *versus* hospital pulmonary rehabilitation, followed by telephone or conventional follow-up. *Health Technol Assess* 2010; 14: i–v, vii–xi, 1–140.

37 Soicher JE, Mayo NE, Gauvin L, *et al.* Trajectories of endurance activity following pulmonary rehabilitation in COPD patients. *Eur Respir J* 2012; 39: 272–278.

38 Heppner PS, Morgan C, Kaplan RM, *et al.* Regular walking and long-term maintenance of outcomes after pulmonary rehabilitation. *J Cardiopulm Rehabil* 2006; 26: 44–53.

39 Chan AW, Lee A, Suen LK, *et al.* Effectiveness of a Tai chi Qigong program in promoting health-related quality of life and perceived social support in chronic obstructive pulmonary disease clients. *Qual Life Res* 2010; 19: 653–664.

40 Yeh GY, Roberts DH, Wayne PM, *et al.* Tai chi exercise for patients with chronic obstructive pulmonary disease: a pilot study. *Respir Care* 2010; 55: 1475–1482.

41 Leung RW, Alison JA, McKeough ZJ, *et al.* Ground walk training improves functional exercise capacity more than cycle training in people with chronic obstructive pulmonary disease (COPD): a randomised trial. *J Physiotherapy* 2010; 56: 105–112.

42 Breyer MK, Breyer-Kohansal R, Funk GC, *et al.* Nordic walking improves daily physical activities in COPD: a randomised controlled trial. *Respir Res* 2010; 11: 112.
