# Supplementary material for: The Effects of Traditional Chinese Exercise in Patients with Chronic Obstructive Pulmonary Disease: A Meta-Analysis
Source: PLoS One. 2016 Sep 2;11(9):e0161564. doi: 10.1371/journal.pone.0161564 (PMC5010221; doi:10.1371/journal.pone.0161564)
Supplement: S2 Appendix — (ZIP) [file pone.0161564.s002.zip › S2 Appendix Reasons for excluded articles/Reasons for excluded articles.docx]

**Reasons for exclusion:**

1. Tai chi mind-body exercise in patients with COPD: study protocol for a randomized controlled trial

**Reason：****It is a study protocol, not a** **randomized controlled trial.**

1. Sun-style T’ai Chi improves walking endurance and health-related quality of life in people with COPD

**Reason：It is a critically appraised paper. This article does not provide complete data. Although we have tried to find ways to search for the data of this article, but did not find it.**

1. Effects of comprehensive therapy based on traditional Chinese medicine patterns in stable chronic obstructive pulmonary disease: a four-center, open-label, randomized, controlled study

**Reason：The method of this article is conventional Western medicine and Bu-Fei Jian-Pi granules, Bu-Fei Yi-Shen granules, and Yi-Qi Zi-Shen granules based on the TCM patterns, not the Traditional Chinese Exercise.**

1. T’ai chi for individuals with COPD: an ancient wisdom for a 21st century disease?

**Reason：It is a review, not a randomized controlled trial.**

1. Benefits and costs of home-based pulmonary rehabilitation in chronic obstructive pulmonary disease: a-multi-centre randomised controlled equivalence trial.

**Reason: The method of this article is home-based pulmonary rehabilitation (e.g.step-ups on an internal or external step, sit to stand from a standard height chair, water bottles for upper limb weights),not Traditional Chinese Exercise included Taichi, Qigong or Liu Zijue or Ba Duanjin or Wu Qinxi or Yi Jinjing.**
